# The COMET Handbook: version 1.0

**DOI:** 10.1186/s13063-017-1978-4

**Published:** 2017-06-20

**Authors:** Paula R. Williamson, Douglas G. Altman, Heather Bagley, Karen L. Barnes, Jane M. Blazeby, Sara T. Brookes, Mike Clarke, Elizabeth Gargon, Sarah Gorst, Nicola Harman, Jamie J. Kirkham, Angus McNair, Cecilia A. C. Prinsen, Jochen Schmitt, Caroline B. Terwee, Bridget Young

**Affiliations:** 1 MRC North West Hub for Trials Methodology Research, Department of Biostatistics, University of Liverpool, Block F Waterhouse Building, 1-5 Brownlow Street, Liverpool, L69 3GL UK; 2 Centre for Statistics in Medicine, Nuffield Department of Orthopaedics, Rheumatology and Musculoskeletal Sciences, University of Oxford, Oxford, UK; 3 MRC ConDuCT II Hub for Trials Methodology Research, School of Social and Community Medicine, University of Bristol, Bristol, UK; 4 Centre for Public Health, Queen’s University Belfast, Belfast, UK; 5 National University of Ireland Galway and HRB Trials Methodology Research Network, Galway, Ireland; 6 Department of Epidemiology and Biostatistics, EMGO+ Institute for Health and Care Research, VU University Medical Center, Amsterdam, The Netherlands; 7 Center for Evidence-based Healthcare, Medizinische Fakultät, Technische Univesität Dresden, Dresden, Germany

**Keywords:** Core outcome set, Clinical trial, COMET Initiative, Patients and the public

## Abstract

The selection of appropriate outcomes is crucial when designing clinical trials in order to compare the effects of different interventions directly. For the findings to influence policy and practice, the outcomes need to be relevant and important to key stakeholders including patients and the public, health care professionals and others making decisions about health care. It is now widely acknowledged that insufficient attention has been paid to the choice of outcomes measured in clinical trials. Researchers are increasingly addressing this issue through the development and use of a core outcome set, an agreed standardised collection of outcomes which should be measured and reported, as a minimum, in all trials for a specific clinical area.

Accumulating work in this area has identified the need for guidance on the development, implementation, evaluation and updating of core outcome sets. This Handbook, developed by the COMET Initiative, brings together current thinking and methodological research regarding those issues. We recommend a four-step process to develop a core outcome set. The aim is to update the contents of the Handbook as further research is identified.

**Electronic supplementary material:**

The online version of this article (doi:10.1186/s13063-017-1978-4) contains supplementary material, which is available to authorized users.

## Chapter 1: Background

### 1.1 Outcomes in clinical trials

Clinical trials are research studies undertaken with human beings for the purpose of assessing the safety and effectiveness of interventions, treatments or care procedures. Randomised controlled trials (RCTs) are seen as the ‘gold standard’ in evaluating the effects of treatments [1].

There are three basic components of randomised clinical trials [2]:

1. At least one test treatment and a comparator treatment
2. Randomisation of treatment allocation
3. Outcome measure(s)

It is the third component that is the focus of this Handbook. Broadly, in the context of clinical trials, an outcome is defined to be a measurement or observation used to capture and assess the effect of treatment such as assessment of side effects (risk) or effectiveness (benefits). When designing a clinical trial, the ‘PICO’ format is often used to formulate a research question. A ‘well-built’ question should include four parts; that is identifying the patient problem or population (P), the intervention (I), the comparator (C) and the outcomes of interest (O) [3]. In a randomised trial, differences between the groups in outcomes can be inferred to be as a result of the differing interventions. Therefore, the selection, measurement and reporting of important, relevant and appropriate outcomes are critical.

Researchers, clinicians and policy-makers often distinguish between the *efficacy* and the *effectiveness* of an intervention. Whereas efficacy trials (also described as explanatory trials) determine whether an intervention can have a beneficial effect in an ideal situation under optimum conditions [4], effectiveness trials (also described as pragmatic trials) measure the degree of beneficial effect under ‘real-world’ clinical settings. In contrast to an efficacy trial, an effectiveness trial will usually be conducted following as close to clinical practice as possible [5]. Design of effectiveness trials are, therefore, based on conditions of, and with consideration to, routine clinical practice and clinical decision-making. Efficacy trials tend to precede effectiveness trials, and although it is preferential to distinguish between efficacy and effectiveness trials, in reality they exist on a continuum [1], often making it difficult to separate the two as distinct phases of research. The focus of this Handbook will be effectiveness trials.

Clinical trials will usually include multiple outcomes of interest, and the main outcomes are usually those essential for decision-making. Some outcomes will be of more interest than others. The primary outcome is typically chosen to be the one of greatest therapeutic importance [6] to relevant stakeholders, such as patients and clinicians, is an integral component of the research question under investigation and is usually the one used in the sample size calculation [7]. Sometimes, researchers propose more than one primary outcome if they are thought to be of equal therapeutic importance and relevance to the research question. This can also be useful if it is unclear which single primary outcome will best answer the question. Secondary outcomes evaluate other beneficial or harmful effects of secondary importance or are useful for explaining additional effects of the intervention [8]. Secondary outcomes may also be exploratory in nature. Harmful effects should always be viewed as important regardless of their primary or secondary outcome label [7]. In addition to assessing relative therapeutic benefit and safety, decision-makers are usually also interested in the acceptability and cost-effectiveness of the interventions under study.

A variety of different types of outcomes can be measured in trials, and researchers must decide which of these to measure. As well as the importance of an outcome to relevant stakeholders, researchers must consider an array of information, including how responsive it is to the interventions being compared and the appropriateness to the trial; for example, the financial cost and acceptability to patients associated with measuring that outcome. The decision is made more complex by the numerous types of outcomes that exist, and researchers must decide which of these types of outcomes is most appropriate for both the question under investigation and the specific context of the clinical trial. For example, a clinical outcome describes a medical event(s) that occurs as a result of disease or treatment [9], and relates to a patient’s symptoms, overall mental state or how the patient functions. In contrast, a surrogate endpoint is used as a substitute for a clinical outcome [10]. A biomarker is another type of outcome and is a medical sign, typically used in earlier phase trials, used to predict biological processes. Examples of biomarkers include everything from pulse and blood pressure through basic chemistries to more complex laboratory tests of blood and other tissues [11].

In addition to deciding what to measure, Zarin et al. describe that a fully specified outcome measure includes information about the following [12]: domain (e.g. anxiety), that is *what* to measure; specific measurement (e.g. Hamilton Anxiety Rating Scale), that is *how* to measure that outcome/domain; the specific metric used to characterise each participant’s results (e.g. change from baseline at specified time); and method of aggregation (e.g. a categorical measure such as proportion of participants with a decrease greater than 50%).

Furthermore, outcomes can also be measured in different ways. Some clinical outcomes are composed of a combination of items, and are referred to as composite outcomes. Outcomes can be objective, that is not subject to a large degree of individual interpretation, and these are likely to be reliably measured across patients in a study, by different health care providers, and over time. Laboratory tests may be considered objective measures in most cases. Outcomes may also be considered to be subjective. Most clinical outcomes involve varying degrees of subjectivity; for example, a diagnosis or assessment by a health care provider, carer or the patient themselves. A clinician-reported outcome is an assessment that is determined by an observer with some recognised professional training that is relevant to the measurement being made. In contrast, an observer-reported outcome is an assessment that is determined by an observer who does not have a background of professional training that is relevant to the measurement being made, i.e. a non-clinician observer such as a teacher or caregiver. This type of assessment is often used when the patient is unable to self-report (e.g. infants, young children). Finally, a patient-reported outcome is a measurement based on a report that comes directly from the patient (i.e. the study participant) about the status of particular aspects of or events related to a patient’s health condition [9].

### 1.2 Problems with outcomes

Clinical trials seek to evaluate whether an intervention is effective and safe by comparing the effects of interventions on outcomes, and by measuring differences in outcomes between groups. Clinical decisions about the care of individual patients are made on the basis of these outcomes, so clearly the selection of outcomes to be measured and reported in trials is critical. The chosen outcomes need to be relevant to health service users and others involved in making decisions and choices about health care. However, a lack of adequate attention to the choice of outcomes in clinical trials has led to avoidable waste in both the production and reporting of research, and the outcomes included in research have not always been those that patients regard as most important or relevant [13].

Inconsistencies in outcomes have caused problems for people trying to use health care research, illustrated by the following two examples [14]: (1) a review of oncology trials found that more than 25,000 outcomes appeared only once or twice [15] and (2) in 102/143 (71%) Cochrane reviews, the authors were unable to obtain the findings for key outcomes in the included trials, with 26 (18%) missing data for the review’s prespecified primary outcome from over half of the patients included in the research [16]. In addition, variability in how outcomes are defined and measured can make it difficult, or impossible, to synthesise and apply the results of different research studies. For example, a survey of 10,000 controlled trials involving people with schizophrenia found that 2194 different measurement scales had been used [17].

Alongside this inconsistency in the measurement of outcomes, outcome-reporting bias adds further to the problems faced by users of research who wish to make well-informed decisions about health care. Outcome-reporting bias has been defined as the selection of a subset of the original recorded outcomes, on the basis of the results, for inclusion in the published reports of trials and other research [18]. Empirical evidence shows that outcomes that are statistically significant are more likely to be fully reported [19]. Selective reporting of outcomes means that fully informed decisions cannot be made about the care of patients, resource allocation, research priorities and study design. This can lead to the use of ineffective or even harmful interventions, and to the waste of health care resources that are already limited [20].

### 1.3 Standardising outcomes

#### 1.3.1 Core outcome sets (COS)

These issues of inconsistency and outcome-reporting bias could be reduced with the development and application of agreed standardised sets of outcomes, known as core outcome sets (COS), that should be measured and reported in all trials for a specific clinical area [21]. As previously noted [22], these sets represent ‘the minimum that should be measured and reported in all clinical trials of a specific condition and could also be suitable for use in other types of research and clinical audit’ [23]. It is to be expected that the core outcomes will always be collected and reported, and that researchers will likely also include other outcomes of particular relevance or interest to their specific study. Measuring a COS does not mean that outcomes in a particular trial necessarily need to be restricted to just those in the set.

The first step in the development of a COS is typically to identify *what* to measure [22]. Once agreement has been reached regarding what should be measured, *how* the outcomes included in the core set should be defined and measured is then determined.

The use of COS will lead to higher-quality trials, and make it easier for the results of trials to be compared, contrasted and combined as appropriate, thereby reducing waste in research [22]. This approach would reduce heterogeneity between trials because all trials would measure and report the agreed important outcomes, lead to research that is more likely to have measured relevant outcomes due to the involvement of relevant stakeholders in the process of determining what is core, and be of potential value to use in clinical audit. Importantly, it would enhance the value of evidence synthesis by reducing the risk of outcome-reporting bias and ensuring that all trials contribute usable information.

#### 1.3.2 Core outcome set initiatives

One of the earliest examples of an attempt to standardise outcomes is an initiative by the World Health Organisation in the 1970s, relating to cancer trials [24]. More than 30 representatives from groups doing trials in cancer came together, the result of which was a WHO Handbook of guidelines recommending the minimum requirements for data collection in cancer trials. The most notable work to date relating to outcome standardisation since has been conducted by the OMERACT (Outcome Measures in Rheumatology) collaboration [25] which advocates the use of COS, designed using consensus techniques, in clinical trials in rheumatology. This, and other relevant initiatives, is described below.

OMERACT is an independent initiative of international health professionals interested in outcome measures in rheumatology. The first OMERACT conference on rheumatoid arthritis was held in Maastricht, in the Netherlands in 1992 [26]. The motivation for this was discussions between two of the executive members, comparing the outcomes for patients with rheumatoid arthritis in European clinical trials with that of North American clinical trials, and noting that they used different endpoints. This made it extremely difficult to compare and combine in meta-analyses. Over the last 20 years, OMERACT has served a critical role in the development and validation of clinical and radiographic outcome measures in rheumatoid arthritis, osteoarthritis, psoriatic arthritis, fibromyalgia, and other rheumatic diseases. OMERACT strives to improve outcome measurement in rheumatology through a ‘data driven’, iterative consensus process involving relevant stakeholder groups [27].

An important aspect of OMERACT now is the integration of patients at each stage of the OMERACT process, but this was not always the case. Initially, OMERACT did not include patients in the process of developing COS. The patient perspective workshop at OMERACT 6 in 2002 addressed the question of looking at outcomes from the patient perspective. Fatigue emerged as a major outcome in rheumatoid arthritis, and it was agreed that this should be considered for inclusion in the core set [28–30]. This patient input along with clinical trialist insight, epidemiologist assessment and industry perspective, has led OMERACT to be a prominent decision-making group in developing outcome measures for all types of clinical trials and observational research in rheumatology. OMERACT has now developed COS for many rheumatological conditions, and has described a conceptual framework for developing core sets in rheumatology (described in ‘Section 0’) [31].

Since OMERACT there have been other examples of similar COS initiatives to develop recommendations about the outcomes that should be measured in clinical trials. One example is the Initiative on Methods, Measurement, and Pain Assessment in Clinical Trials (IMMPACT) [32], whose aim is to develop consensus reviews and recommendations for improving the design, execution and interpretation of clinical trials of treatments for pain. The first IMMPACT meeting was held in November 2002, and there have been a total of 17 consensus meetings on clinical trials of treatments for acute and chronic pain in adults and children. Another exemplar is the Harmonising Outcome Measures for Eczema (HOME) Initiative [33]. This is an international group working to develop core outcomes to include in all eczema trials.

#### 1.3.3 The COMET Initiative

The COMET (Core Outcome Measures in Effectiveness Trials) Initiative brings together people interested in the development and application of COS [34]. COMET aims to collate and stimulate relevant resources, both applied and methodological, to facilitate exchange of ideas and information, and to foster methodological research in this area. As previously described [35], specific objectives are to:

1. Raise awareness of current problems with outcomes in clinical trials
2. Encourage COS development and uptake
3. Promote Patient and Public Involvement (PPI) in COS development
4. Provide resources to facilitate these aims
5. Avoid unnecessary duplication of effort
6. Encourage evidence-based COS development

The COMET Initiative was launched at a meeting in Liverpool in January 2010, funded by the MRC North West Hub for Trials Methodology (NWHTMR). More than 110 people attended, with representatives from trialists, systematic reviewers, health service users, clinical teams, journal editors, trial funders, policy-makers, trials registries and regulators. The feedback was uniformly supportive, indicating a strong consensus that the time was right for such an initiative. The meeting was followed by a second meeting in Bristol in July 2011 which reinforced the need for COS across a wide range of areas of health and the role of COMET in helping to coordinate information about these. COMET has gone on to have subsequent successful international meetings in Manchester (2013), Rome (2014) [36] and Calgary (2015) [37] to affirm this.

For COS to be an effective solution, they need to be easily accessible by researchers and other key groups. Previously, it has been difficult to identify COS because they are hard to find in the academic literature. This might mean that they have not been used in new studies or that there has been unnecessary duplication of effort in developing new COS. The COMET Initiative sought to tackle this problem by undertaking a systematic review of COS (see section ‘Populating the COMET database’), and to reduce the possibility of waste in research by bringing these resources together in one place. The COMET website and database were launched in August 2011. The COMET Initiative database is a repository of studies relevant to the development of COS. In addition to the searchable database, the website provides:

- Information about COMET, including aims and objectives and a description of the COMET Management Group
- Information about upcoming and past COMET events, including workshops and meetings organised by the COMET Initiative
- Resources for COS developers, including relevant publications, examples of grant-funded projects, examples of COS development protocols, plain language summaries and PPI resources
- Relevant web links, including core outcome networks and collaborations, patient involvement, how to measure, and research funding

The growing awareness of the need for COS is reflected in the website and database usage figures [22, 38]. Use of the website continues to increase, with more than 20,900 visits in 2015 (25% increase over 2014), 15,366 unique visitors (25% increase), 13,049 new visitors (33% increase) and a rise in the proportion of visits from outside the UK (11,090 visits; 53% of all visits). By December 2015, a total of 9999 searches had been completed, with 3411 in 2014 alone (43% increase).

##### 1.3.3.1 Populating the COMET database

A systematic review of COS was conducted in August 2013 [14], and subsequently updated to include all published COS up to, and inclusive of, December 2014 [39]. The aim of the systematic reviews was to identify studies which had the aim of determining which outcomes or domains to measure in all clinical trials in a specific condition, and to identify and describe the methodological techniques used in these studies. The two reviews have identified a total of 227 published COS up to, and including, December 2014. The systematic reviews highlighted great variability in the ways that COS had been developed, particularly the methods used and the stakeholders included as participants in the process. The update demonstrated that recent studies appear to have adopted a more structured approach towards COS development and public representation has increased. We will be referring to the results of these reviews in more detail throughout the Handbook.

As noted above, the types of studies included in the database are those in which COS have been developed, as well as studies relevant to COS development, including systematic reviews of outcomes and patients’ views. Individuals and groups who are planning or developing a COS, who have completed one or who have identified one in an ad hoc way can submit it for inclusion. There are now over 120 ongoing COS included in the COMET database (correct as of April 2016). Furthermore, we maintain a separate list of studies (not included in the database), to include information on studies that have not yet progressed beyond an expression of interest or a very early stage in their development. It also contains some studies where permission is needed before they can be included in the main, online database. This list currently includes approximately 90 studies (correct as of February 2016).

#### 1.3.4 Other relevant initiatives

Whilst the initiatives described above are specific to the development of COS for trials in particular areas of health, there are a few other recent initiatives relevant to the improvement of outcome measurement. One such initiative is the Core Outcomes in Women’s Health (CROWN) initiative. This is an international group, led by journal editors, to harmonise outcome reporting in women’s health research [40]. This consortium aims to promote COS in the specialty, encourage researchers to develop COS and facilitate reporting of the development of COS.

The US National Institutes of Health (NIH) encourages the use of common data elements (CDEs) in NIH-supported research projects or registries. The NIH provide a resource portal that includes databases and repositories of data elements and Case Report Forms that may assist investigators in identifying and selecting data elements for use in their projects [41]. PROMIS is another NIH initiative and is part of the NIH goal to develop systems to support NIH-funded research supported by all of its institutes and centres [41]. PROMIS provides a system of measures of patient-reported health status for physical, mental health and social health which can be used across chronic conditions (see description in ‘Ontologies for grouping individual outcomes into outcome domains’ in Chapter 2). Once it has been decided what outcomes should be measured, PROMIS is a source of information regarding how those outcomes could be measured.

Once a COS has been agreed, it is then important to determine how the outcomes included in the set should be defined and measured. Several measurement instruments may exist to measure a given outcome, usually with varying psychometric properties (e.g. reliability and validity). Important sources of information for selecting a measurement instrument for a COS are systematic reviews of measurement instruments. The COnsensus-based Standards for the selection of health Measurement INstruments (COSMIN) initiative collates systematic reviews of measurement properties of available measurement instruments that intend to measure (aspects of) health status or (health-related) quality of life. An overview of these reviews and guidelines for performing such reviews can be found on the COSMIN website [42]. COSMIN has also developed a checklist about which measurement properties are important and standards for how to evaluate their measurement properties [43]. The COSMIN checklist will facilitate the selection of the most appropriate PRO measure amongst competing instruments. A collaboration between COSMIN and COMET has recently resulted in the development of a guideline on how to select outcome measurement instruments for outcomes included in a COS [44]. COSMIN has recently started a new project with the aim of developing a separate version of the COSMIN checklist for non-PRO measurements.

The Clinical Outcome Assessments (COA) staff at the US Food and Drug Administration (FDA), previously known as the Study Endpoints and Labelling Development (SEALD) Study Endpoints Team, aim to encourage the development and application of patient-focussed endpoint measures in medical product development to describe clinical benefit in labelling. The COA staff engage with stakeholders to improve clinical outcome measurement standards and policy development, by providing guidance on COA development, validation and interpretation of clinical benefit endpoints in clinical trials. The FDA define a COA as a ‘measure of patient’s symptoms, overall mental state, or the effects of a disease or condition on how the patient functions’. Put simply, the COA staff work to ensure that the evidence provided about an outcome instrument can be relied upon in the context of drug development and regulatory decision-making.

The Clinical Data Interchange Standards Consortium (CDISC) is a global Standards Development Organisation (SDO) ‘to develop and support global, platform-independent data standards that enable information system interoperability to improve medical research and related areas of health care’ [45]. CDISC aims to establish worldwide industry standards to support the electronic acquisition, exchange, submission and archiving of clinical research data and metadata to improve data quality and streamline medical and biopharmaceutical product development and research processes. The Coalition for Accelerating Standards and Therapies (CFAST) Initiative is a CDISC partnership, set up to accelerate clinical research and medical product development by creating and maintaining data standards, tools and methods for conducting research in therapeutic areas that are important to public health [45]. One of its objectives is to identify common standards for representing clinical data for drug studies in priority therapeutic areas. This includes standardising definitions of outcomes, and the way in which outcomes are described.

### 1.4 The aim of this Handbook

As stated above, one of the aims of the COMET Initiative is to encourage evidence-based COS development. Although some guidance exists for COS developers [21, 46, 47], it is varied in the level of detail and is often unclear regarding the level of evidence available to support particular methodological recommendations.

The aim of the Handbook is to describe current knowledge in the area of COS development, implementation, review and uptake, and to make recommendations for practice and research as appropriate. The Handbook will be updated as further research is undertaken and reported, in order to continue to inform good methodological practice in this area.

**Fig. 1:**
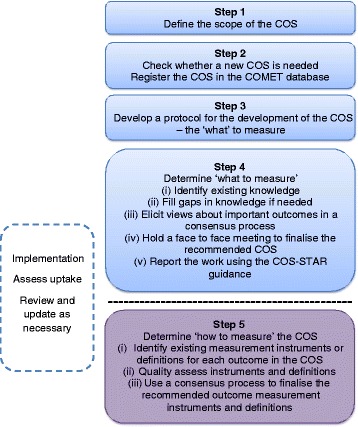
The core outcome set (COS) development process

Figure 1 illustrates the approach taken to COS development in this Handbook. Steps 1–4 are covered in detail in Chapter 2, and describe a process for determining what to measure, the COS. Step 5, determining how to measure the outcomes in the COS, is covered briefly since much has been written and described elsewhere. Step 5 is crucial to achieve future uptake and thereby realise the benefits of COS development. Chapter 3 covers implementation, review and feedback. Chapter 4 provides recommendations for practice, proposes a methodological research agenda, and discusses other areas where the use of COS may be beneficial.

## Chapter 2: Developing a core outcome set

### 2.1 Background

The development of a COS in health care involves working with key stakeholders to prioritise large numbers of outcomes and achieve consensus as to the core set. Various methods have been used to develop a COS and it is uncertain which are most suitable, accurate and efficient. Research to identify optimal methods of developing COS is ongoing and there is currently wide variation in the approaches used [14]. Methods include the Delphi technique [48, 49], nominal group technique [50, 51], consensus development conference [52] and semistructured group discussion [53]. Many studies have used a combination of methods to reach consensus’ for example, Ruperto et al. [54] used the Delphi approach followed by the nominal group technique at a face-to-face meeting, whilst Harman et al. [55], Potter et al. [56], van’t Hooft et al. [57] and Blazeby et al. [58] used the Delphi approach followed by face-to-face semistructured discussion.

One example where consensus work has been undertaken in two different ways is in paediatric asthma. The American Thoracic Society/European Respiratory Society employed an expert panel approach [59], whereas other researchers combined results from a Delphi survey with clinicians and interviews with parents and children [60]. The results were overlapping but not identical. Female sexual dysfunction is another disease area where different methods have been used to obtain consensus. In one study, a literature review was undertaken and critiqued by experts [61], whereas in another study, a modified Delphi method was used to develop consensus definitions and classifications [62]. Both studies resulted in the same primary outcome; however, secondary outcomes differed. Similarly, multiple COS have also been developed for systemic lupus erythematosus. OMERACT adopted a nominal group process to rank outcome domains [63], whereas EULAR adopted a consensus building approach [64]. The results from both studies were very similar, with EULAR recommending other additional outcomes.

COS developers have identified that methodological guidance for COS development would be helpful [65]. There is limited empirical evidence, however, regarding whether different methods lead to similar or different conclusions, and there is a need to develop evidence-based approaches to COS development.

The OMERACT Handbook is a useful resource for those wishing to develop COS in the area of rheumatology under the umbrella of the OMERACT organisation [46]. We have previously identified issues to be considered in the development of COS more generally [21] and expand on those here, together with additional ones identified since this earlier publication. We present information about how COS developers have tackled these issues using data from our previous systematic reviews [14, 39] and describe results from methodological research studies where available.

In the systematic review (227 COS identified), 63% of studies made recommendations about what to measure only. Some of the remaining studies also made recommendations about how to measure the outcomes that they included in their core set, with 35% of studies doing this as a single process, considering both what to measure and how to measure. The remaining 2% of studies in the systematic review of COS considered what to measure and how to measure outcomes included in the core set as a two-stage process, first considering what to measure and then considering how to measure. Thus, there appears to be consistency in that the first step in the process is typically to gain agreement about ‘what’ to measure, with decisions about ‘how’ and ‘when’ to measure these outcomes usually later in the process. This two-stage process has the advantage of being able to identify gaps where further research would be needed, e.g. if an outcome is deemed to be of core importance but no outcome measurement instrument exists with adequate psychometric properties.

This chapter provides guidance on developing consensus about *what* to measure, i.e. a COS, and provides recommendations for finding and selecting instruments for measuring the outcomes in the core set, i.e. the *how*.

### 2.2 Scope of a core outcome set

The scope of a COS refers to the specific area of health or health care of interest to which the COS is to be applied. The scope should be described in terms of the health condition, target population and interventions that the COS is to be applicable to, thus covering the first three elements of the PICO (Population, Intervention, Comparator, Outcomes) structure for a clinical trial.

This can be one of the most difficult aspects of the process, but clarity from the outset will likely reduce later problems of misinterpretation and ambiguity. This will help to focus the development of the COS and help potential users decide on its relevance to their work.

#### 2.2.1 Health condition and target population

For example, in prostate cancer, a COS may be developed for all patients or it may focus on patients with localised disease.

#### 2.2.2 Interventions

For example, a COS may be created for use in all trials of interventions to treat localised prostate cancer or just for surgery.

Of the 227 COS published up to the end of 2014, 53% did not specify whether the COS was intended for all interventions or a particular intervention type, 7% were for any intervention, and 40% were for a specific intervention type.

#### 2.2.3 Setting

The focus of this Handbook is on the development of COS for effectiveness trials. A distinction is made between efficacy and effectiveness trials, since developing a COS to cover both designs may lead to difficulties with respect to particular domains such as health care resource use [48]. COS are equally applicable in other settings; for example, routine clinical practice (see ‘Chapter 4’).

### 2.3 Establishing the need for a core outcome set

#### 2.3.1 Does a relevant core outcome set already exist?

The first thing to do is find out whether a relevant COS exists by reviewing the academic literature.

One of the difficulties in this area of research has been to identify whether studies have already been done, or are underway, to develop a COS. The COMET Initiative has developed an online searchable database, enabling researchers to check for existing or ongoing work before embarking on a new project, thus minimising unnecessary duplication of effort. A video of ‘How to search the COMET database’ can be found on the COMET website [66].

The COMET database is populated through an annual systematic review update of published studies, and by COS developers registering their new projects. To avoid missing any ongoing projects not yet registered in the COMET database, it is recommended that researchers contact other experts in the particular health condition, as well as the COMET project coordinator, to check whether any related work is ongoing. It may also be prudent to apply the COMET search strategy [14] with additional filter terms for the area of interest for the recent period since the last COMET annual update.

Although there may be no exact match for the scope of interest, it may be that a related COS exists, e.g. a COS for all interventions in the condition of interest has been developed but a COS for a specific intervention type is sought, or a COS was developed by relevant stakeholders in countries other than that of the team with the current interest, or a COS was developed with the same scope but did not involve obtaining patients’ views.

#### 2.3.2 Is a core outcome set needed?

If a relevant COS does not exist, a review of previous trials [67] or systematic reviews [68] in the area can provide evidence of need for a COS. Systematic reviewers are starting to use the outcome matrix recommended by the ORBIT project [69] to display the outcomes reported in the eligible studies. This matrix may demonstrate inconsistency of outcomes measured to date in addition to potential outcome-reporting bias.

The rest of this chapter is written from the premise that the development of a new COS is warranted. If a COS already exists, but the quality could be improved by additional work related to particular stakeholder groups, countries, or alternative consensus methods, then certain sections below will also be of relevance. The issue of quality assessment is discussed in ‘Quality assessment/critical appraisal’ below and in ‘Chapter 4’.

#### 2.3.3 Avoiding unnecessary duplication of effort

The COMET database is a useful resource for researchers to see what work has been done in their area of interest and for research funders wishing to avoid unnecessary duplication of effort when supporting new COS activities, as illustrated by the following two examples.

##### Example 1

In September 2014 Valerie Page (Watford General Hospital, UK) contacted COMET via the website to register the development of a COS in delirium. We followed up the request for additional information so that we could register this in the database, and in the meantime we logged this on the private non-database list that we use to keep track of work that we know about prior to inclusion in the database. Whilst waiting for this information to be returned, in May 2015 we received a second request for registration of COS development in the same clinical area by Louise Rose from the University of Toronto, Canada. The researchers were unaware of each other’s work. We got in touch with both researchers and asked for permission to share details of their work, as well as to pass on contact details. In September 2015 we received confirmation that Louise Rose and Valerie Page, with the European Delirium Association and American Delirium Society, are now working collaboratively on this. Details of this collaborative effort to develop a COS for delirium can be found in the database [70].

##### Example 2

Benjamin Allin (University of Oxford, UK) started planning a study to develop a COS for infants with gastroschisis in early 2015. He checked the COMET database to see if a COS existed, but nothing was registered at that time. He contacted COMET in September 2015 to register his project. On receiving this request, the COMET project coordinator checked the COMET database to find out if there was any relevant work in this area and identified an ongoing study registered in this same area of gastroschisis. This latter work had been registered by Nigel Hall (University of Southampton and Southampton Children’s Hospital, UK) in June 2015. Again, the two groups were put in touch, and they met up to discuss the proposed core sets, which resulted in a plan being drawn up for collaboration to work together to produce one COS rather than two. The existing gastroschisis COS entry in the database has been updated to reflect this collaborative effort [71].

### 2.4 Study protocol

There are potential sources of bias in the COS development process, and preparing a protocol in advance may help to reduce these biases, improve transparency and share methods with others. We recommend that a protocol be developed prior to the start of the study, and made publically available, either through a link on the COMET registration entry or a journal publication [72–74]. In a similar way to the development of the SPIRIT guidance for clinical trial protocols, there is a need to agree protocol content.

### 2.5 Project registration

One of the aims of the COMET Initiative is to provide a means of identifying existing, ongoing and planned COS studies. COS developers should be encouraged to register their project in a free-to-access, unrestricted public repository, such as the COMET database, which is the only such repository we are aware of.

The following information about the scope and methods used is recorded in the database for existing and ongoing work:

1. Clinical areas for which the outcomes are being considered, identifying both primary disease and types of intervention
2. Target population (age and sex), and any other details about the population within the health area
3. Setting for intended use (e.g. research and/or practice)
4. Method of development to be used for the COS
5. People and organisations involved in identifying and selecting the outcomes, recording how the relative contributions will be used to define the COS

Details of any associated publications, including the protocol and the final report, can be recorded in the COMET database, added to the original COMET registration page.

### 2.6 Stakeholder involvement

It is important to consider which groups of people should be involved in deciding which outcomes are core to measure, and why. Bringing diverse stakeholders together to try to reach a consensus is seen to be the future of collaborative, influential research.

Key stakeholders may include health service users, health care practitioners, trialists, regulators, industry representatives, policy-makers, researchers and the public. Decisions regarding the stakeholder groups to be involved, how they are to be identified and approached, and the number from each group will be dependent upon the particular scope of the COS as well as upon existing knowledge, the methods of COS development to be used, and practical feasibility considerations. For example, a COS for an intervention that aims to improve body image, e.g. breast reconstruction following mastectomy, is likely to have predominantly patients as the key stakeholders [56].

The stages of involvement during the process should also be considered for each stakeholder group. For example, it may be considered appropriate to involve methodologists in determining how to measure particular outcomes, but not to be involved in determining what to measure. These decisions should be documented and explained in the study protocol.

Consideration should be given to the representativeness of the sample of stakeholders and the ability of people across the different groups to engage with the chosen consensus method (including online activities and face-to-face meetings).

Consideration should be given to potential conflicts of interest within the group developing the COS (for example, the developers of measurement instruments in the area of interest or those whose work is focussed on a specific outcome).

#### 2.6.1 Patient and public involvement and participation

COMET recognises the expertise and crucial contribution of patients and carers in developing COS. COS need to include outcomes that are most relevant to patients and carers, and the best way to do this is to include them in COS development. Examples exist where patients have identified an outcome important to them as a group that might not have been considered if the COS had been developed by practitioners on their own [75, 76]. However, it is worth noting that examples also exist where health professionals have identified areas that patients were reluctant to talk about in focus groups; for example, sexual health [77].

##### 2.6.1.1 Patient and public participation

We refer to patients taking part in the COS study as ‘research participants’ and the activity as research ‘participation’. People involved in a COS study as research participants give their views on the importance of outcomes and may also subsequently be asked their opinion on how those outcomes are to be measured.

Of the 227 COS that had been published up to the end of December 2014, 44 (19%) studies reported including patient participants in the COS development process. However, of these 44 COS, only 26 (59%) studies provided details of how patients had participated in the development process. The most commonly used methods to include patient participants were the Delphi technique and semistructured group discussion which were used in 38% and 35% of studies, respectively. Three of the 26 (12%) COS studies were developed with only patients as participants. Of the remaining 23 studies, patients participated alongside clinicians during the development process in 19 (83%) studies, as compared to two (9%) studies where patients and clinicians participated separately throughout the whole development process. In the two remaining studies, patients and clinicians participated separately in the initial stages, but then alongside side each other during the final stages of the development process. For the 21 studies where patients and clinicians did participate alongside each other for all or part of the COS development process, the percentage of patient participants included ranged from 4 to 50%.

Of ongoing COS studies (n = 127 as of 12 April 2016), 88% now include patients as participants. The question now is not whether patients should participate, but rather the nature of that participation. It is recommended that both health professionals and patients be included in the decision-making process concerning what to measure, as the minimum, unless there is good reason to do otherwise. ‘Qualitative methods in core outcome set development’ below discusses considerations to enhance patient participation in a COS.

##### 2.6.1.2 Patient and public involvement

When planning a COS study that involves patients as research participants, it is important to also involve patients in designing the study. We refer to patients who are involved in designing and overseeing a COS study as ‘public research partners’ and this activity as ‘patient involvement’. PPI has been defined as where research is ‘being carried out “with” or “by” members of the public rather than “to”, “about” or “for” them’ [78].

Involving public research partners in both the design and oversight of the COS development study may have the potential to:

- Provide advice on the best ways of identifying and accessing particular patient populations
- Inform discussions about ethical aspects of the study
- Facilitate the design of more appropriate study information
- Promote the development of more relevant materials to promote the study
- Enable ongoing troubleshooting opportunities for patient participation issues during the study, e.g. recruitment and retention issues of study participants
- Inform the development of a dissemination strategy of COS study results for patient participants and the wider patient population
- Ensure that your COS is relevant to patients and, crucially, that patients see it to be relevant and can trust that the development process has genuinely taken account of the patient perspective.

Involving public research partners in designing and overseeing the COS study requires that researchers plan for this involvement. They might choose different methods of doing this; for example, they might have one or two discussion groups in the planning stage and then ongoing involvement of one or two public contributors on the Study Advisory Group (SAG). For example, Morris et al. (2015) engaged parents at various stages of the research process and consulted with parents from their ‘Family Faculty’ in designing a plain language summary of the results of their COS [79]. Numerous resources now exist to help researchers to plan and budget for PPI in research; for example: in the UK, INVOLVE have numerous resources [80].

COMET has also produced a checklist for COS developers to consider with public research partners when planning their COS study. These can be found on the COMET website.

### 2.7 Determining ‘what’ to measure – the outcomes in a core outcome set

#### 2.7.1 Identifying existing knowledge about outcomes

It is recommended that potential relevant outcomes are identified from existing work to inform the consensus process. There are three data sources that should be considered: systematic reviews of published studies, reviews of published qualitative work, investigation into items collected in national audit data sets and interviews or focus groups with key stakeholders to understand their views of outcomes of importance. Depending on the resources available, protocols within clinical trial registries may also be a useful source of information.

##### 2.7.1.1 Systematic review of outcomes in published studies

Systematic reviews are advantageous because they can efficiently identify an inclusive list of outcomes being reported by researchers in a given area. Nevertheless, it is important to note that systematic reviews of outcomes just aggregate the opinions of the previous researchers on what outcomes they deemed important to measure; hence the need for subsequent consensus development to agree with the wider community of stakeholders what outcomes should be included in a COS.

The scope of the systematic review should be carefully considered in the context of the COS to ensure that outcomes are included from all relevant studies without unnecessary data collection. The clinical area should be clearly defined and appropriate databases accessed accordingly. Commonly used databases include Medline, CINAHL, Embase, the Cochrane Database of Systematic Reviews and PsycINFO. In the systematic reviews of COS [14, 39], 57 (25%) studies carried out a review of outcomes [65]. The number of databases searched was not reported for 17 studies (30%), and two studies did not perform an electronic database search. Thirty-eight studies described which databases they searched (Table 1).

**Table 1:** Description of databases searched (*n* = 38)

Number of databases	*n*	Databases searched
1	18	Medline (*n* = 9)
		PubMed (*n* = 7)
		Central Register of Controlled Trials (*n* = 2)
2	8	Medline and Embase (*n* = 4)
		PubMed and Central Register of Controlled Trials (*n* = 1)
		Medline and CancerLit (*n* = 1)
		Medline and Central Register of Controlled Trials (*n* = 1)
		Medline and PubMed (*n* = 1)
3	4	Medline, CINAHL and Embase (*n* = 2)
		Medline, Embase and Cochrane Central Register of Controlled Trials (*n* = 1)
		PubMed, CINAHL, PsychINFO (*n* = 1)
4	1	PubMed, Medline, Embase and the Cochrane Collaboration
5	3	Medline, PreMedline, CancerLit, PubMed (National Library of Medicine) and Cochrane Library (*n* = 1)
		Medline, Embase, PsycINFO, Cochrane Library and CINAHL (*n* = 1)
		Cochrane Wounds Group Specialised Register, The Cochrane Central Register of Controlled Trials (CENTRAL) (The Cochrane Library), Medline, Embase and CINAHL (*n* = 1)
6	0	
7	2	Cochrane Oral Health Group’s Trials Register (CENTRAL), Medline, Embase, Science Citation Index Expanded, Social Science Citation Index, Index to Scientific and Technical Proceedings, System for Information on Grey Literature in Europe (*n* = 1)
		Cochrane Skin Group Specialised Register, the Cochrane Central Register of Controlled Trials in The Cochrane Library (Issue 4, 2009), Medline, Embase, AMED, PsycINFO, LILACS (*n* = 1)
8	2	CINAHL (Cumulative Index to Nursing and Allied Health Literature), Embase, Medline, National Criminal Justice Reference Service (NCJRS), PsycINFO, Sociological Abstracts, The Cochrane Database, The Patient-reported Health Instruments (PHI) website (*n* = 1)
		Medline, PubMed, Embase, PsycINFO, CINAHL, Web of Sciences, Cochrane Central Register of Controlled Trials, and Cochrane Database of Systematic Reviews (*n* = 1)

There is no recommended time window to conduct systematic reviews. Some COS studies may examine all the available academic literature. This may be an enormous task in common disease areas. Scoping searches are useful to determine the number of identified studies for a specific area. Overly large reviews are resource intensive and may not yield important additional outcomes. One strategy is to perform the systematic review in stages to check if outcome saturation is reached. For example, a review of trials published over the last 5 years may be conducted initially and the outcomes extracted. The search may then extended, and the additional outcomes checked against the original list. If there are no further outcomes of importance then the systematic review may be considered complete. For most areas a recent search is recommended as a minimum (e.g. the past 24 months) to capture up-to-date developments and outcomes relevant to that COS. Seventeen studies in the systematic reviews of COS (30%) did not state the date range searched. Seven studies (12%) did not apply any date restrictions to their search. The number of years reported in the remaining 33 studies ranged between 2 and 59. Frequencies are provided in Table 2.

**Table 2:** Number of years searched (*n* = 33)

Number of years searched	Frequency
Less than 5	1
5 to 9	3
10 to 14	12
15 to 19	2
20 to 24	6
25 to 29	1
30 to 34	1
35 to 39	4
More than 40	3

Data extraction should be considered in terms of:

1. Study characteristics
2. Outcomes
3. Outcome measurement instruments and/or definitions provided by the authors for each outcome

In terms of outcome extraction from the academic literature, it is recommended that all are extracted verbatim from the source manuscript [81]. This transparency is important to allow external critical review of the COS right back to its inception. In addition, extraction of outcome definitions supplied by, and measurement instruments used by, the authors is recommended as this will inform the selection of the outcome measurement set which will occur at a later stage. This is necessary because outcome definitions may vary widely between investigators and it is often not clear as to what outcomes are measuring [67, 82–84].

##### 2.7.1.2 How to extract outcomes from the academic literature to inform the questionnaire survey

It is likely that some outcomes will be the same but will have been defined or measured in different publications in various ways. For example, in a review of outcomes for colorectal cancer surgery some 17 different definitions were identified for ‘anastomotic leakage’ [85]. The first step is to group these different definitions together (extracting the wording description verbatim) under the same outcome name. Similarly, in a review of outcomes for weight loss surgery, it was apparent that different terminology is used for weight loss itself in the academic literature [84]. The 41 different outcome assessments referring to weight were all categorised into one item for a subsequent Delphi questionnaire survey.

The next step is to group these outcomes into outcome domains, constructs which can be used to classify broad aspects of the effects of interventions, e.g. functional status. Outcomes from multiple domains may be important to measure in trials, and several outcomes within a domain may be relevant or important. Initially researchers create outcome domains for each outcome to be grouped into (see ‘Ontologies for grouping individual outcomes into outcome domains’ below). The domains need discussion and to be agreed by the team for the list to be categorised. Each outcome will then be mapped to a domain (independently) and this will provide transparency. For example, in a systematic review of studies evaluating the management of otitis media with effusion in children with cleft palate, a total of 43 outcomes were listed under 13 domain headings (see Table 18 in [81]).

Categorisation of each verbatim outcome definition to an outcome name, and each outcome name to an outcome domain is recommended to be performed independently by two researchers from multiprofessional backgrounds. This may include expert health service researchers, clinicians (e.g. surgeons, dietician, nurses, health psychologists) and methodologists. Where two researchers work on this process a senior researcher will need to resolve differences and make final decisions.

##### 2.7.1.3 Systematic review of studies to identify outcomes of importance to health service users

Similarly, it is necessary to systematically review the academic literature to identify Patient-reported Outcome Measures (PROMs) and then extract patient-reported outcome domains. These come from existing PROMs often at the level of the individual questionnaire item [86]. This is recommended because the scale name used in PROMs and the scores attributed to the combined items are often found to be inconsistent. Therefore, analyses at a granular level are recommended [86]. The full process for this is described in Fig. 1 of the paper by Macefield et al. At this stage it is worth extracting details of the patient-reported outcome development and validity which will be helpful when selecting measures with which to assess the core outcomes.

A PRO long list extracted from PROMs may be supplemented with additional domains derived from a review of qualitative research studies if time allows (e.g. [87, 88]). It is recommended that interpretation of data from qualitative papers is guided by experts in the field.

#### 2.7.2 Identifying and filling the gaps in existing knowledge

It is important to identify which key stakeholder groups’ views are not encompassed by systematic reviews of outcomes in published studies or the existing academic literature more generally, and decide whether these are gaps that need to be filled. An initial list from published clinical studies may be supplemented by undertaking qualitative research with key stakeholders whose views are important yet unlikely to be represented within systematic reviews of outcomes in previous studies. Where resources are limited, consultation with an advisory group whose membership reflects the key stakeholders may be used as an alternative to qualitative research, but it should be noted that such consultation is not qualitative research and the information arising from it does not have the same standing as the knowledge generated by research.

Qualitative interviews or focus groups with key stakeholders, especially patients, are recommended, particularly if the PROMS have lacked detailed patient participation in their development. The following section outlines in more detail how qualitative work may contribute to COS development. Nevertheless, it is recommended that qualitative research is guided by researchers with expertise in these. Interviews should be performed with a purposeful sample and use a semistructured interview schedule to elicit outcomes of importance to that population. The interview schedule may be informed by the domain list generated from the academic literature or be more informed by a grounded theory approach and start with very open questions. Interviews are audio-recorded, transcribed and analysed for content. The information can then be used to create new outcome domains or supplement the long list [89, 90].

#### 2.7.3 Ontologies for grouping individual outcomes into outcome domains

Outcome domain models or frameworks exist to attempt to provide essential structure to the conceptualisation of domains [91], and have been used to classify outcomes that have been measured in clinical trials in particular conditions. Despite their intended use to provide a framework, there is not always consistency between the different models. In a review of Health-related Quality of Life (HRQoL) models, Bakas et al. found that there were wide variations in terminology for analogous HRQoL concepts [91]. Outcome hierarchies have been proposed for specific conditions [92] and cancer [93].

There have been several frameworks to classify health, disease and outcomes to date. There are various conceptual frameworks relevant to outcomes in health and these cover somewhat different areas of outcomes, some of which are described below.

##### 2.7.3.1 Outcome-related frameworks

**World Health Organisation (WHO)**The WHO definition of health, although strictly a definition of health, can be considered a framework as it includes three broad health domains [94]: physical, mental and social wellbeing. This definition has not been amended since 1948 but is a useful starting place to study health. In a scoping review of conceptual frameworks, Idzerda et al. point out that although the three domains are clearly outlined, no further information about what should be included within each domain is provided [95].

##### Patient-reported Outcomes Measurement Information System (PROMIS)

The PROMIS domain framework builds on the WHO definition of health to provide subordinate domains beneath the broad headings stated above [41]: physical (symptoms and functions), mental (affect, behaviour and cognition) and social wellbeing (relationships and function). It was developed for adult and paediatric measures as a way of organising outcome measurement tools.

##### World Health Organisation International Classification of Functioning Disability and Health (WHO ICF)

The International classification of Functioning, Disability and Health (ICF) offers a framework to describe functioning, disability and health in a range of conditions. The ICF focuses on the assessment of an individual’s functioning in day-to-day life. It provides a framework for body functions, activity levels and participation levels in basic areas and roles of social life; providing domains of biological, psychological, social and environmental aspects of functioning [96]. In many clinical areas, ICF core sets have been developed. These core sets identify the most relevant ICF domains for a particular health condition.

##### 5Ds

5Ds is presented as a systematic structure for representation of patient outcomes and includes five ‘dimensions’: death, discomfort, disability, drug or therapeutic toxicity, and dollar cost [97]. This representation of patient outcome was developed specifically for rheumatic diseases, and the authors claim that each dimension represents a patient outcome directly related to patient welfare; for example, they describe how a patient with arthritis may want to be alive, free of pain, functioning normally, experiencing minimal side effects and be financially solvent. This framework assumes that outcomes are multidimensional, and it is critical that the ‘concept of outcome’ is orientated to patient values.

##### Wilson and Cleary

Wilson and Cleary [98] propose a taxonomy or classification for different measures of health outcome. They suggest that one problem with other models is the lack of specification about how outcomes interrelate. They divide outcomes into five levels: biological and physiological factors, symptoms, functioning, general health perceptions, and overall quality of life. In addition to classifying these outcome measures, they propose specific causal relationships between them that link traditional clinical outcomes to measures of health-related quality of life. For example, ‘Characteristics of the environment’ are related to ‘Social and psychological supports’ which in turn relates to ‘Overall quality of life’. Ferrans et al. [99] revised the Wilson and Cleary model to further clarify and develop individual and environmental factors.

##### Outcome Measures in Rheumatology (OMERACT) Filter 2.0

The OMERACT Filter 2.0 [31] is a conceptual framework that encompasses ‘the complete content of what is measurable in a trial’. That is, a conceptual framework of measurement of health conditions in the setting of interventions. It comprises three core areas: death, life impact and pathophysiological manifestations; it also comprises one strongly recommended, resource use. These core areas are then further categorised into core domains. They liken the areas to ‘large containers’ for the concepts of interests (domains and subdomains). They recommend that the ICF domains are also considered under life impact (ICF domains: activity and participation) and pathophysiological manifestations (ICF domains: body function and structure). Although OMERACT recommends the inclusion in a COS of at least one outcome reflecting each core area, empirical evidence is emerging that this is not always considered appropriate [48].

##### Outcome Measures Framework (OMF)

The Outcome Measures Framework (OMF) project was funded by the Agency for Healthcare Research and Quality (a branch of the U.S. Department of Health and Human Services) to create a conceptual framework for development of standard outcome measures used in patient registries [100]. The OMF has three top-level broad domains: characteristics, treatments and outcomes. There are six subcategories within the outcomes domain: survival, disease response, events of interest, patient/caregiver-reported outcomes, clinician-reported outcomes and health system utilisation. The model was designed so that it can be used to define outcome measures in a standard way across medical conditions. Gliklich et al. conclude that ‘as the availability of health care data grows, opportunities to measure outcomes and to use these data to support clinical research and drive process improvement will increase’.

##### Survey of Cochrane reviews

Rather than attempting to define outcome domains as others have done, Smith et al. performed a review of outcomes from Cochrane reviews to see whether there were similar outcomes across different disease categories, in an attempt to manage and organise data [101]. Fifteen categories of outcomes emerged as being prominent across Cochrane Review Groups and encompassed person-level outcomes, resource-based outcomes, and research/study-related outcomes. The 15 categories are: adverse events or effects (AE), mortality/survival, infection, pain, other physiological or clinical, psychosocial, quality of life, activities of daily living (ADL), medication, economic, hospital, operative, compliance (with treatment), withdrawal (from treatment or study) and satisfaction (patient, clinician, or other health care provider). The authors recognise that these 15 categories might collapse further.

##### 2.7.3.2 Use of outcome-related frameworks in core outcome set studies

In the systematic reviews of COS [14, 39], 17 studies provided some detail about how outcomes were grouped or classified (Table 3).

**Table 3:** Methods for classifying/grouping outcomes (*n* = 17)

Reference	Method for classifying/grouping outcomes
Duncan (2000) [225]	Each outcome measure was classified into one of the following categories: death or, at the level of pathophysiological parameters (blood pressure, laboratory values, and recanalisation), impairment, activity, or participation. Measures were classified according to the system used by Roberts and Counsell [226], which includes the Rankin/modified Rankin scale as a measure of activity rather than participation
Sinha (2012) [60]	Each outcome was grouped into one of the following six outcome domains, some of which were further divided into subdomains: disease activity, physical consequence of disease, functional status, social outcomes and quality of life, side effects of therapy and health resource utilisation. Where it was unclear which domain was appropriate, this was resolved by discussion between the authors. Reference given in support of this approach: Sinha et al. 2008 [227]
Broder (2000) [129]	List developed by staff at institution (but no further detail)
Distler (2008)[228]	The results of this literature search were discussed at the first meeting of the Steering Committee. Based on this discussion, a list of 17 domains and 86 tools was set up for the first stage of the Delphi exercise to define outcome measures for a clinical trial in PAH-SSc. Domains were defined as a grouping of highly related features that describe an organ, disease, function, or physiology (e.g. cardiac function, pulmonary function, and quality of life)
Devane (2007) [173]	Outcome measures addressing similar dimensions or events were discussed by the team and collapsed where possible. For example, various modes of delivery/birth were presented as ‘mode of birth (e.g. spontaneous vaginal, forceps, vaginal breech, caesarean section, vacuum extraction)’. This pilot tool was tested for clarity, with a sample of 12 participants, including 3 maternity care consumers, and subsequently refined
Smaïl-Faugeron (2013) [111]	Because we expected a large diversity in reported outcomes, we grouped similar outcomes into overarching outcome categories by a small-group consensus process. The group of experts consisted of 6 dental surgeons specialising in paediatric dentistry, including 3 clinical research investigators. First, the group identified outcomes that were identical despite different terms used across trials. Second, different but close outcomes (i.e. outcomes that could be compared across studies or combined in a meta-analysis) were grouped together into outcome domains. Finally, the group, with consensus, determined several outcome categories and produced a reduced-outcome inventory
Merkies (2006) [229]	In advance of the workshop, a list of outcome measures applied in treatment trials was prepared including their scientific soundness, WHO and quality of life classification (WHO classification reference is ICF)
Rahn (2011) [230]	From this outcome inventory, the outcomes were organised and grouped into eight proposed overarching outcome domains: 1. Bleeding; 2. Quality of life; 3. Pain; 4. Sexual health; 5. Patient satisfaction; 6. Bulk-related complaints; 7. Need for subsequent surgical treatment and 8. Adverse events. Categories were determined based on their applicability to all potential interventions for abnormal uterine bleeding and the physician expert group’s consensus of their relevance for informing patient choices. Outcomes related to cost, resource use, or those determined by the review group to have limited relevance for assessing clinical effectiveness were excluded from categorisation and further analyses
Chow (2002) [231]	Some detail but process not described – the endpoints employed in previous bone metastases trials of fractionation schedules were identified and listed in the first consensus survey under the following headings: 1. Pain assessments; 2. Analgesic assessments and primary endpoint; 3. Endpoint definitions; 4. Timing, frequency and duration of follow-up assessment; 5. When to determine a response; 6. Progression and duration of response; 7. Radiotherapy techniques; 8. Co-interventions following radiotherapy; 9. Re-irradiation. 10. Non-evaluable patients (lost follow-up) and statistics; 11. Other endpoints; 12. Other new issues and suggestions and 13. Patient selection issues
Van Der Heijde (1997) [232]	Grouped into patient-assessed, physician-assessed or physician-ordered measures
Chiu (2014) [233]	A wide variety of different outcomes measures were reported [in the studies included in the systematic review]. We classified these into 4 categories: postoperative alignment, sensory status, control measures and long-term change
Fong (2014) [139]	Twenty-one maternal and 24 neonatal outcomes were identified by our systematic reviews, one randomised controlled trial and two surveys. The maternal components included complications associated with pre-eclampsia and were broadly classified under neurological, respiratory, haematological, cardiovascular, gastrointestinal, renal and other categories. The neonatal components were prematurity-associated complications involving respiratory, neurological, gastrointestinal, cardiovascular systems and management-based outcomes such as admission to the neonatal unit, inotropic support and use of assisted ventilation
Fraser (2013) [234]	Based on an overall evaluation of intra-arterial head and neck chemotherapy, there are several outcome variables that should be monitored and reported when designing future trials. These outcome variables can be categorised into ‘Procedure-related’, ‘Disease control’ and ‘Survival’
Goldhahn (2014) [235]	The group reached an agreement to use the ICF thereby identifying key domains within this framework using the nominal group technique. Recently, the WHO has established core sets for hand conditions [236]. A Comprehensive Core Set of 117 ICF categories were selected appropriate for conducting a comprehensive, multidisciplinary assessment. A brief core set of 23 ICF categories were selected, and considered more appropriate for individual health care professionals. The body functions contained in this core sets include emotional function, touch function sensory functions related to temperature and other stimuli, sensation of pain, mobility of joint functions, stability of joint functions, muscle power functions, control voluntary movement functions, and protective functions of the skin. The group agreed that the ICF categories would be consistent with clinicians’ current practice patterns of focussing on pain, joint range of motion and hand strength [237]
Saketkoo (2014) [238]	The Delphi process began with an ‘item-collection’ stage called tier 0, wherein participants nominated an unrestricted number of potential domains (qualities to measure) perceived as relevant for inclusion in a hypothetical 1-year RCT. This exercise produced a list of >6700 items—reduced only for redundancy, organised into 23 domains and 616 instruments and supplemented by expert advisory teams of pathologists and radiologists
Smelt (2014) [159]	We then grouped the answers [from round 1 of the Delphi questionnaires] according to the presence of strong similarity. During this process, we followed an inductive method, i.e. answers were examined and those considered to be more or less the same were grouped as one item. No fixed number of items was set beforehand in order to accommodate all new opinions. The answers were grouped by two of the authors (AS and VdG) separately, to ensure independence of assessments. Any discrepancies were resolved through a discussion with two other authors (ML and DK) who also checked whether they agreed with the items as formulated by AS and VdG
Wylde (2014) [137]	The pain features that were retained after the three rounds of the Delphi study were reviewed and systematically categorised into core outcome domains by members of the research team [86]. The IMMPACT recommendations [239] were used as a broad framework for this process. Each individual feature was reviewed to determine whether it was appropriate to group it into an IMMPACT-recommended pain outcome (pain intensity, the use of rescue treatments, pain quality, temporal components of pain) or a new pain outcome domain. The developed outcomes domains were then reviewed to ensure that the features that they encompassed adequately reflected the domain and that the features were conceptually similar. These core outcome domains were subsequently discussed and refined by the Project Steering Committee and the PEP-R group

*ICF* International classification of Functioning, Disability and Health, *PAH-SSc* pulmonary arterial hypertension associated with systemic sclerosis, *PEP-R* Patient Experience Partnership in Research (a patient and public involvement group specializing in musculoskeletal research), *WHO* World Health Organisation

##### 2.7.3.3 Use of outcomes recommended in core outcome sets to inform an outcome taxonomy

Based on the classification of outcomes in two previous cohorts of Cochrane systematic reviews [101, 102] and the outcomes recommended in 198 COS [14], the following taxonomy has been proposed:

1. Mortality
  Includes subsets all, cause-specific, quality of death, etc.
2. Physiological (or Pathophysiological)
  Disease activity (e.g. cancer recurrence, asthma exacerbation, includes ‘physical consequence of disease’, etc.)
  Blood pressure, laboratory values, recanalisation
3. Infection
  New, recurrent
4. Pain
5. Quality of life
  Includes Health-related Quality of Life (HRQoL)
6. Mental health
7. Psychosocial (includes behavioural)
8. Function (or Functional status)
  Does this cover activities? Participation? (Read the Roberts and Counsell paper referenced in the review of stroke outcomes)
9. Compliance with/withdrawal from treatment
10. Satisfaction
  Reported by patient, health professional, etc.
11. Resource use (or health resource utilisation)
  Includes subset hospital, community, additional treatment, etc.
12. Adverse events (or side effects)
  Be clear that this could include things like death, pain, etc. when they are unanticipated harmful effects of an intervention

Pilot work is underway with selected Cochrane Review Groups to test the taxonomy for applicability. To date, one additional outcome domain, knowledge, has been identified as missing from the list.

#### 2.7.4 Determining inclusion and wording of items to be considered in the initial round of the consensus exercise

It is important to spend time on this aspect of the process, in terms of the structure, content and wording of the list of items, to avoid imbalance in the granularity of item selection and description and ambiguity of language. Participants in the consensus process may identify such issues, necessitating revisions to the list during subsequent rounds [48]. A SAG (see ‘Achieve global consensus’ below) can provide valuable input at the design stage, prior to the start of the formal consensus process.

The review of existing knowledge, and research to fill gaps in that knowledge, has the potential to result in a long list of items. Consideration is needed regarding whether to retain the full list in the consensus exercise or whether to reduce the size of the list using explicit criteria. Preparatory work on how best to explain the importance of scoring all items on the list may help to improve levels of participation.

As noted in the section on qualitative research in COS development, because qualitative research involves patients and other stakeholders describing their views and experiences in their own terms, it gives COS developers access to the words, phrases and language that patients use to describe how conditions or interventions affect them. COS developers can, therefore, incorporate the words that patients use in interviews and focus groups to label and explain outcome items in a Delphi, thereby ensuring that the items are understandable and accessible for patients. Pilot or pretesting work involving cognitive or ‘think aloud’ interviews to examine how patients and other stakeholders interpret the draft items can help to refine the outcome labels and explanations [103, 104]. As the name suggests, this technique literally involves asking participants to think aloud as they work through the draft Delphi and provide a running commentary on what they are thinking as they read the items and consider their responses. This allows COS developers to understand the items from the perspective of participants. Cognitive interviews are widely used in questionnaire development to refine instruments and ensure they are understandable for the target groups.

Other methods previously used to determine the description of items include a reading-level assessment and amendment as necessary [55], and a review of terminology used in existing health frameworks such as ICF [96], PROMIS [41], the Wilson and Cleary model [98] as well as related COS [48].

#### 2.7.5 Short- or longer-term outcome assessment

One issue to consider is whether, and how, to address the timing of outcome assessment. Many COS developers have identified an agreed set of outcomes to measure, leaving the timing of assessment as an issue for trialists to decide subsequently depending on their particular context of use. In an alternative approach, in the COS for rheumatoid arthritis [26] agreed at a face-to-face meeting, it is recommended that radiological damage is only measured in trials where the patients are to be followed up for longer than 1 year. It is recommended that the approach to handling this issue be made clear to participants from the outset in the subsequent consensus process, to avoid ambiguity later on.

#### 2.7.6 Eliciting views about important outcomes

Having identified a list of potential outcomes, the next step is to assess the level of importance given to each. Considerations concerning the choice of assessment method include the need to build a consensus with methodological rigor, and to adopt strategies to ensure that a diverse range of opinions is heard.

Methods used in previous studies to elicit opinions and to develop consensus about important outcomes include expert panel meetings (sometimes using nominal group technique (NGT) methods) and Delphi surveys. A single, heterogeneous consensus panel comprising the various stakeholders may be deemed appropriate for particular areas of health care whereas separate panels for different stakeholder groups followed by work to integrate the multiple perspectives may be more appropriate for others.

If participants in a consensus process are shown a list of potential outcomes, we recommend that in general they should be given the opportunity to propose the inclusion of additional items, especially as the academic literature may not include outcomes associated with the most recent treatments available or the most pressing current concerns for stakeholders.

We consider a Delphi exercise to be a useful way of gaining information about opinion from a wide group of participants. Of 127 ongoing COS in the COMET database (as of 12 April 2016), 108 (85%) involve a Delphi survey, and hence we discuss this method in more detail below.

##### 2.7.6.1 The Delphi technique

With the exception of the Delphi technique, all other methods for COS development described earlier involve face-to-face communication. The Delphi technique is advantageous in that it is anonymous, avoiding the effect of dominant individuals, and can be circulated to large numbers with wide geographic dispersion.

The Delphi technique was originally developed by Dalkey and Helmer (1963) at the Rand Corporation in the 1950s [105]. In a COS framework, the method is used for achieving convergence of opinion from experts (stakeholders) on the importance of different outcomes in sequential questionnaires (or rounds) sent either by post or electronically. Responses for each outcome are summarised and fed back anonymously within the subsequent questionnaire. Participants are able to consider the views of others before re-rating each item and can, therefore, change their initial responses based on the feedback from the previous rounds. With no direct communication between participants this feedback provides a mechanism for reconciling different opinions of stakeholders and is, therefore, critical to achieving consensus.

There remains, however, uncertainty as to the optimum way to use such methodology. Many issues need to be considered at the outset, all of which may have an impact on the final results. These include:

1. Number of panels
2. Group size
3. Participant information
4. Number of rounds
5. Structure of the questionnaires
6. Methods of scoring
7. Nature of feedback presented between rounds
8. Criteria for retaining outcomes between rounds
9. Attrition (response bias) between rounds
10. Consensus definitions
11. How the degree of consensus will be assessed

In the following sections, we discuss each of the above issues in detail and offer guidance on different approaches.

##### Single or multiple panels for different stakeholders

The choice of stakeholder groups to be involved in the development of a COS has been discussed in ‘Stakeholder involvement’ in Chapter 2 above. There may be additional considerations regarding which groups should be involved in a Delphi survey. For example, in a COS for early stage dementia, interviews rather than Delphi survey participation may be considered to be the more appropriate way to include patient views.

What also requires consideration is how best to combine the views of different stakeholders within a Delphi survey. The issue of the impact of panel composition on Delphi performance has seldom been investigated in general [106]. Some COS studies have used a single panel of experts from one particular stakeholder group or combined a heterogeneous group of participants, representing multiple stakeholder groups, into a single panel (that is, ignoring stakeholder status when generating feedback and assessing consensus). Others have used multiple homogenous panels, each formed by a different stakeholder group. In rheumatology, Ruperto et al. [54] used a single panel of paediatric rheumatologists whilst developing a COS in systemic lupus erythematosus (JSLE) and juvenile dermatomyositis (JDM), whilst Taylor et al. [107] combined the views of rheumatologists and industry representatives into a single panel in the development of a COS in chronic gout. The MOMENT study (Management of Otitis Media with Effusion in Cleft Palate) considered eight separate stakeholder groups and treated them as multiple separate panels [55].

The single homogeneous panel approach will result in core outcomes deemed essential by only one stakeholder group. If a single panel is formed by combining heterogeneous stakeholder groups (such that feedback and criteria for consensus are based on the group overall and ignore stakeholder type), careful consideration and justification is needed of the panel mix. If the data are simply amalgamated with no consideration of the separate stakeholder groups, the resulting set may depend on the relative proportions of stakeholders participating or on weightings that may be used for different groups. As an example of imbalance in stakeholder representation, the Taylor et al. study [107] had only three industry representatives and the remaining 26 respondents were rheumatologists. The single-panel approach here is clearly in favour of the rheumatologists’ opinions.

In areas where differing stakeholder opinions are expected, a better approach would be to consider multiple panels, retaining distinct stakeholder groups when generating feedback and considering criteria for consensus (see later sections). The final core set, or outcomes taken forward to the next stage of COS development, may then consist of (1) outcomes deemed essential by all stakeholder groups or (2) outcomes deemed essential by any stakeholder group. The former option may, therefore, result in the most important outcomes for any particular group being excluded from the core set which may not be acceptable. At the same time, whilst including items deemed essential by any relevant stakeholder group ensures that outcomes essential to any group are included, the resulting set may be too extensive to be practical and it could be argued that in this scenario consensus has not been achieved since there will be items that not all groups agreed on. Alternative approaches will be described in a later section on defining consensus (see ‘Defining consensus’ below).

##### Group size

The decision regarding how many individuals to include in a Delphi process is not based on statistical power and is often a pragmatic choice. For example, the group size may be dependent on the number of experts or patients available within the scope of the COS being developed. These numbers may be particularly small if the condition is rare or the intervention of interest is not widely used. In their international Delphi study, Smith and Betts (2014) included only 12 acupuncturists working in pregnancy with at least 5 years’ experience in traditional Chinese medical techniques [108]. As a contrast, Ruperto et al. (2003) enrolled 174 paediatric rheumatologists from two professional organisations in an international Delphi survey [54]. Blazeby et al. (2015) recruited 185 patients and 126 consultants and specialist nurses in a UK-based study to identify a COS for surgery for oesophageal cancer [58], whilst van’t Hooft et al. (2015) involved 32 parents and 163 health professionals in an international Delphi survey as part of the development of a COS for the prevention of preterm birth [57].

Consideration should be given to the number of participants that are invited into the Delphi (allowing for attrition between rounds; see later). Dependent on the consensus definition, the results may be particularly sensitive with smaller numbers of participants. When potential numbers are small, stakeholder group members could be pooled, particularly if it is expected that opinions are unlikely to differ. Typically, such a decision should be done in consultation with the Steering Advisory Group to ensure the appropriateness of the grouping, and without knowledge of the results. Any revisions to the Delphi protocol should be documented with reason.

The key consideration with group size is that there should be good representation from key stakeholder groups with qualified experts who have a deep understanding of the issues. The more participants representing each stakeholder group the better, both in terms of the COS being generalisable to future patients and in convincing other stakeholders of its value.

##### Participant information

It is important for all participants to be fully aware of the purpose of the Delphi survey and what will be expected of them. This is crucial both in terms of enabling informed consent and equipping participants to be able to prioritise and score outcomes. The notion of a COS and even an outcome may not be clear to all. Participant Information Sheets may need to use different terminology for different stakeholder groups and should be piloted in advance. Plain language summaries for patients and carers, including a description of an outcome, a COS and a Delphi survey, are available on the COMET Initiative website [109].

It is also advisable to ensure that the instructions provided within each round of the Delphi survey reiterate the overall aim of achieving consensus of a *core* set of outcomes.

##### Number of rounds

A Delphi survey must consider at least two rounds (that is, at least one round of feedback) to be considered a Delphi survey. The number of Delphi rounds varies across different COS development studies. Typically, COS studies contain two [56, 58, 60, 110] or three rounds [55, 107, 111]. One study reported six rounds [112]; however, this included several rounds of open-ended questions to generate debate in controversial areas in the field of infant spasms and West syndrome. Open-ended rounds may also be used to generate an initial list of outcomes prior to any outcome scoring [60, 113, 114], as an alternative to reviewing the academic literature for example.

Rather than pre-determining the number of rounds, the process can be dynamic with subsequent rounds incorporated if further prioritisation is warranted. Whilst we would not expect, nor require, consensus to be reached on all outcomes in the Delphi questionnaire, it is necessary that a reduced number of outcomes has been agreed (in terms of prespecified criteria) to be of most importance, in order to inform the COS. Outside COS development work, Custer et al. (1999) have recommended that three iterations are sufficient to collect the relevant information to reach a consensus in most cases [115].

From a practical perspective, the number of rounds may also be limited by time, cost or consideration of the burden on participants completing multiple rounds of Delphi. The time taken for participants to complete a round of Delphi is highly variable and will often depend on the number of outcomes being scored. It is advisable to pilot the questionnaires beforehand to ensure that it is practical. Typically, each round of Delphi will remain open for about 2 or 3 weeks, although latter rounds may be kept open longer if response rates are low to try to minimise the potential for attrition bias (see later). Following the closure of a Delphi round, an additional 2 or 3 weeks is required to analyse the data and set up the next round, although this will depend on the design and can be much shorter if using software developed specifically for online Delphi surveys; for example, the DelphiManager software developed by COMET [116].

##### Structure of the questionnaires

Careful consideration is needed when designing the Delphi questionnaire, as for any questionnaire. For example, outside of COS development Moser and Kalton [117] recommend that jargon and technical terms should be avoided in questionnaires; anecdotal evidence from the piloting of Delphi questionnaires for core sets for cancer surgery and OME with cleft palate suggest that lay terms are preferred to technical medical terms, even by health professionals. Stakeholder involvement in the design and piloting of the Delphi questionnaire is recommended to ensure that it is accessible, comprehensible and valid.

##### Order of questionnaire items

Previous research, outside of COS development, has demonstrated that the order in which questions are presented in a questionnaire could affect response rates and actual responses to question items [118]. The idea of the ‘consistency effect’, where items are answered in relation to responses to earlier items, has been researched for more than 50 years with recommendations that general questions should precede specific ones [119], and questions should be grouped into topics [120]. It has also been suggested that if there is evidence that respondents have stronger opinions on some items than others these should be placed first [118]. It has been argued that order effects will be greater for interview surveys and minimal for written surveys since participants have longer to respond to items and have the opportunity to look at all items before responding [121], but effects have been observed in written surveys [122].

Within the development of a COS we are only aware of one published abstract reporting the effects of question order. For surgery for oesophageal cancer recent methodological research considered the impact of the ordering of patient-reported and clinical outcomes in the Delphi survey. Participants were randomly allocated to receive a questionnaire with the patient-reported outcomes presented first and the clinical outcomes last, or vice versa. The study found that ordering of outcomes in a Delphi questionnaire may impact on both response rates and actual responses, hence subsequently impacting on the final core set [123]. Further research is needed to better understand potential order effects in the context of COS development. We are aware of an ongoing nested study in which participants are randomised to one of four orderings of the outcomes throughout the Delphi survey [124].

##### Additional open questions

As described previously, there are different methods of identifying an initial long list of outcomes to inform the Delphi survey. Whatever method is employed the initial list may not be entirely exhaustive and there may be added value in including an open question in the round-1 questionnaire to identify additional outcomes. This open question could be placed at the beginning or the end of the questionnaire depending on the intended purpose.

If placed at the beginning of the questionnaire participants might be asked to identify a small number of outcomes that are of most importance to them before they see the outcomes included in the questionnaire. If placed at the end, participants might be asked to list any additional items that they do not feel have been considered in the questionnaire. The former approach will help to ensure that there are no key outcomes that have been omitted, whilst the latter approach will help to ensure that a more exhaustive list of outcomes. Whether included at the beginning or end of the questionnaire, criteria for including additional items in round 2 should be specified in a protocol; for example, any new outcome suggested may be included, alternatively only those new outcomes suggested by two or more respondents might be added.

##### Scoring system

Core outcome set studies have used a variety of different scoring systems to rate outcomes within a Delphi process, although the majority involve a Likert scale. Other methods include the ranking of outcomes [54, 114] and allocation of points (for example, division of 100 points across all outcomes) [110, 114, 125]. The 9-point Likert scoring system where outcomes are graded in accordance to their level of importance is a common method. Typically, 1 to 3 signifies an outcome is of limited importance, 4 to 6 important but not critical, and 7 to 9 critical [55, 126, 127]. This framework is recommended by the Grading of Recommendations Assessment, Development and Evaluation (GRADE) Working Group for assessing the level of importance about research evidence [21, 128]. Others have used similar 9-point systems. For example Potter et al. [56] and Blazeby et al. [58] asked participants to rate the importance of each outcome on a 1–9 scale where 1 was ‘not essential’ and 9 was ‘absolutely essential’. Some studies have also included an ‘unable to score’ category to allow for the fact that some stakeholder group members may not have the level of expertise to score certain outcomes. As an example, in the development of a COS for otitis media with effusion in children with cleft palate, some of the participants from the speech and language therapist stakeholder group chose not to score some of the outcomes related to the more clinical aspects of the condition [55]. Other studies have used four- [129], five- [111] and seven- [107] point Likert scales to score outcomes.

##### Feedback between rounds

In order to increase the degree of consensus amongst participants, differing views need to be reconciled. The mechanism for this within a Delphi process is the feedback presented to participants in subsequent rounds, enabling different opinions to be considered before re-rating an outcome. At the end of each round the results for each outcome are aggregated across participants and descriptive statistics presented (see later in this section). Participants can be encouraged to provide a reason for their scores on individual outcomes, which can be summarised as part of the feedback.

The generation of these descriptive statistics will depend on whether a single panel or multiple panels have been used. In a single-panel study, feedback ignores any distinct stakeholder groups and summarises and presents scores for each outcome for *all* participants involved, hence hiding any disparate views between stakeholders. As described earlier, if there are disparate views the final COS will depend on the relative proportions of stakeholders. Calculation of summary scores could of course be weighted by stakeholder group, but it is difficult to ascertain what weightings should be given and there is no current guidance on this. In addition, recent evidence in the development of COS suggests that patients are more likely than health professionals to rate an outcome as essential; three studies found that the average score awarded to outcomes in the round-1 questionnaire was greater for patients than health professionals [130], so even in a study involving equal numbers of patients and health professionals, patients may be more likely to influence a core set if outcome scores are simply combined across stakeholder groups.

In a multiple panel study there are three possible approaches to providing feedback. Participants might receive an overall average across all stakeholder groups; however, this would be analogous with the single-panel approach. Alternatively, participants could receive feedback from their own stakeholder group only or from all stakeholder groups separately. If participants receive feedback from their own stakeholder group only, whilst this may enable consensus within stakeholder groups, it provides no opportunity for consensus across groups. Ongoing COS projects include nested randomised studies comparing these three approaches [131, 132].

Recent methodological work, including a before/after study [55] and nested randomised trials [130], examined the impact of providing feedback from all stakeholder groups separately compared to feedback from the participant’s own group only. Type of feedback presented did impact on the subsequent scoring of items [55, 130] and the items subsequently retained at the end of the Delphi process [130]. The research also demonstrated that providing feedback to participants from both stakeholder groups improved consensus between stakeholder groups in terms of reduced variability in responses and improved agreement in items to retain at the end of the Delphi process [130].

In some ongoing COS studies, participants are being asked for the reasons that they have changed scores between rounds particularly if the change in score is from critically important to a score of less importance or vice versa [133, 134]. This will enable us to better understand the impact of feedback and help optimise the Delphi.

**Fig. 2:**
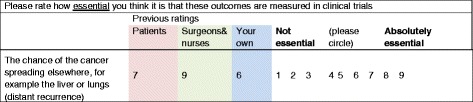
An outcome from a round-2 questionnaire for surgery for colorectal cancer presenting the mean score for round-1 for patients and health professionals separately

**Fig. 3:**
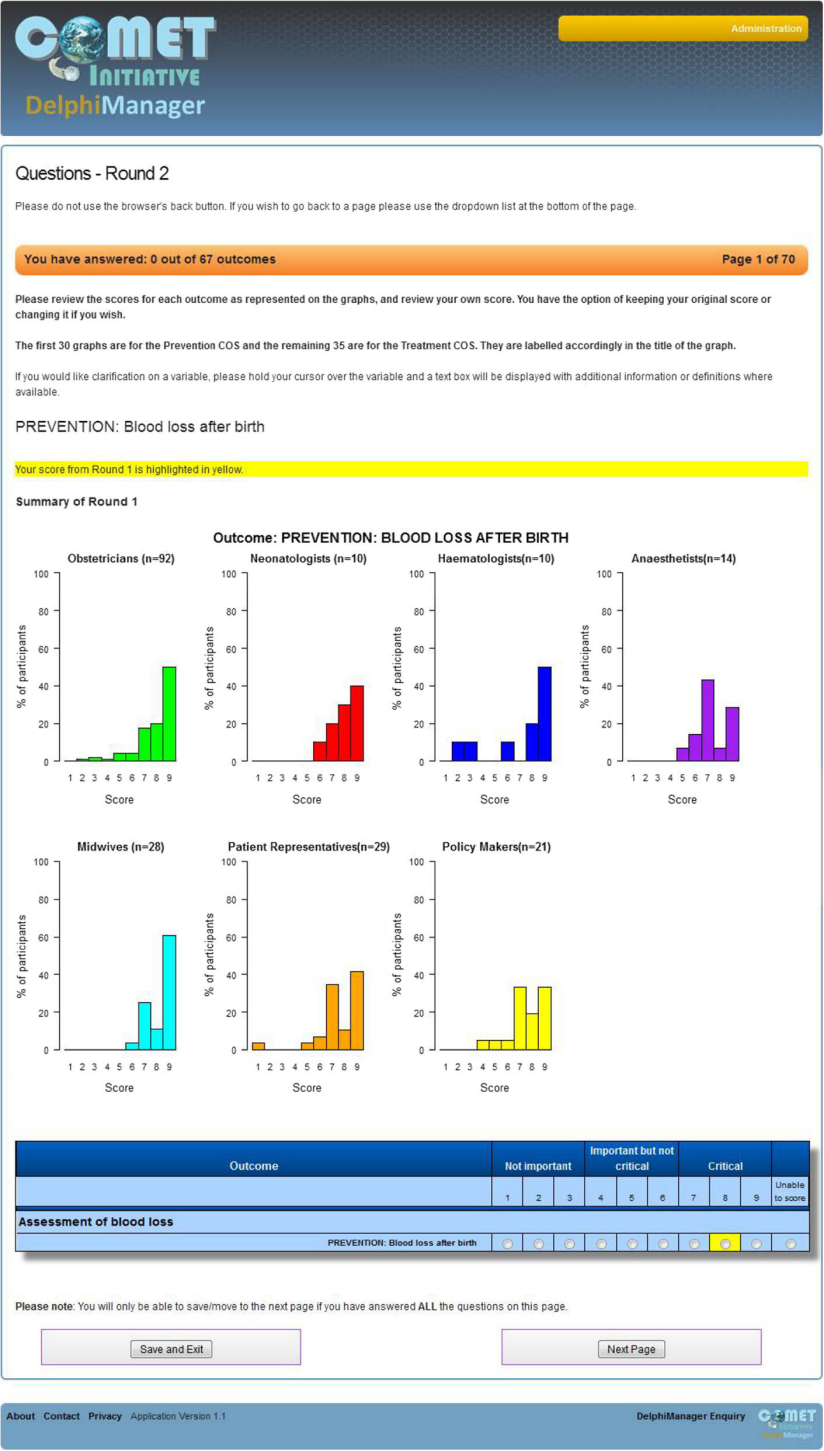
An outcome from a round-2 questionnaire presenting the percentage distribution of scores across all stakeholder groups with options for participants to review their previous round score and re-score (taken from DelphiManager)

There are a number of ways that feedback can be presented. A summary statistic, such as a median or mean (if normally distributed), may be presented for each outcome [58]. Figure 2 demonstrates how feedback was presented in round 2 of a Delphi postal survey used within the development of a COS for surgery for colorectal cancer. Mean scores (rounded to the nearest integer) were presented for both stakeholder groups (patients and surgeons/nurses) included in the study. The participant’s individual score from round 1 is also presented. Single summary statistics are sometimes also presented with a measure of dispersion such as a standard deviation, interquartile range, range or other percentiles [107, 126, 127]. Alternatively, the percentage scoring above a prespecified threshold (for example, 7–9 on a 9-point Likert scale) may be presented [56]; or, the full distribution of scores may be provided graphically. Figure 3 provides a screenshot of an electronic round-2 Delphi questionnaire created within DelphiManager. In this instance a histogram of round-1 scores is presented for each stakeholder group. The participant’s round-1 score is this time highlighted in yellow.

Further research is required to determine which presentation method is most useful and easily interpreted by participants; however, the optimum approach may differ depending on the setting and stakeholder groups involved. Preparatory work with a small group of participant representatives to ensure that the feedback is understood is advisable.

##### Retaining or dropping items between rounds

After the initial Delphi round, subsequent rounds might retain all outcomes [55, 57, 125], or some items may be dropped according to prespecified criteria [56, 58]. Whilst there are examples of both approaches in the academic literature, at present there is no empirical evidence of whether the decision impacts on the final core set. Retaining all items for all rounds may provide a more holistic approach, enabling participants to score and prioritise the list of outcomes as a whole. If items are dropped between rounds there may be items considered of most importance to some participants which are not present in later rounds and this may hinder their ability to prioritise the remaining items. This may be particularly pertinent when scoring systems require participants to allocate a certain number of points across all outcomes [125]. In addition, if items are dropped after the first round, participants will not get the opportunity to re-score those outcomes taking into account feedback on scores from other participants. Suppose that a particular outcome is rated highly by patients in round 1 but poorly by other stakeholder groups and that based on prespecified criteria the outcome is dropped. It is plausible that had participants seen that patients rated the outcome highly, other stakeholders would have increased their scores such that the outcome would have been retained at the end of round 2.

At the same time, if the initial list of outcomes is large, including them in each Delphi round may impose sufficient burden on participants to increase attrition from one round to another. If the decision is made to reduce the number of items from one round to the next, more inclusive criteria for retaining items in earlier rounds may be sensible. For example, in the recent development of a COS for surgery for oesophageal cancer, 67 outcomes were included in round 1. Criteria for inclusion in round 2 were that an item be rated 7 to 9 (on a 9-point Likert scale) by 50% or more participants and 1 to 3 by no more than 15% of participants in at least one stakeholder group [58]. Items were retained at the end of round 2 using stricter cut-off criteria; retained items were rated between 7 and 9 by over 70% of respondents and 1 to 3 by less than 15% by at least one stakeholder group. Using less stringent criteria in earlier rounds, and retaining items for which these criteria are met for any single stakeholder group, reduces the likelihood of dropping outcomes that may have been rated more highly in subsequent rounds had participants been given feedback on them.

In the absence of any empirical evidence to inform the optimum approach, the decision may be largely led by the initial number of outcomes. An intermediate approach, which may to some extent address the disadvantages of both methods, would be to retain all items between rounds 1 and 2, hence enabling participants to re-score in light of feedback for every item, and then drop items in subsequent rounds. Whatever design used, if any items are to be dropped from one round to the next, criteria need to be clearly defined in a protocol.

##### Attrition and attrition bias

The degree of non-response after the first round of the Delphi (attrition) may be highly variable between studies and may be dependent on the timing of Delphi rounds (for example, holiday season may increase attrition), the length of the Delphi (from previous knowledge of completing the previous round), the time elapsed between the first and final round (health care professionals may leave the service, or participants may become disinterested), and the method of recruitment of participants, as well as many other factors. For example, Bennett et al. (2012) observed 0% attrition in their small Delphi study (fewer than 10 participants) but their recruitment strategy was a targeted approach to known experts [126], whereas Smith and Betts (2014) observed higher attrition rates (17%) from 12 participants from inviting trial authors from the relevant academic literature [108]. Similar attrition rates to Smith and Betts were seen in a much larger study for oesophageal cancer surgery which recruited 126 surgeons and nurses identified through a meeting of the Association of Upper Gastrointestinal Surgeons of Great Britain and Ireland, and by personal knowledge of surgeons, and 185 patients recruited from three clinical centres. Attrition rates between rounds 1 and 2 were 15% for professionals and 17% for patients.

If attrition rates are thought to be too high, either overall or for a particular stakeholder group, then strategies should be adopted to increase the response rates. Personalised reminder emails to participants (with details of current response rates), personalised emails from distinguished researchers in the field, direct telephone calls, and the offer of being acknowledged in the study publication have all been found to be helpful strategies in increasing response rates. Consideration should be given to keeping Delphi rounds open longer if it is thought that this may increase response rates. Whilst there is no guidance on what constitutes an acceptable response rate, typically around 80% for each stakeholder group would be deemed satisfactory in most situations.

Attrition bias will occur when the participants that do not respond in subsequent rounds have different views from their stakeholder group peers who continue to participate. For example, if the feedback a participant receives suggests that they are in a minority with regard to their scoring of importance about particular outcomes, then they may be more likely to drop out, leading to over-estimation of the degree of consensus in the final results [135].

**Fig. 4:**
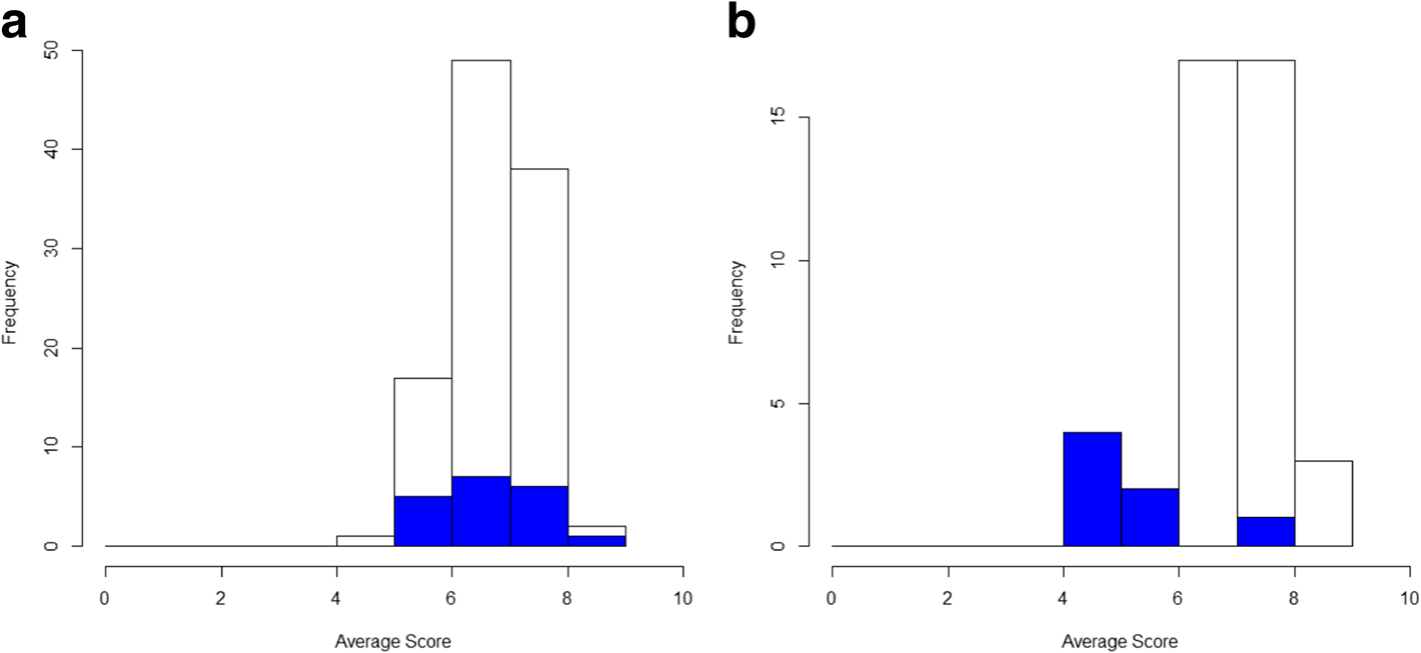
Average scores in round 1 across all outcomes for (**a**) stakeholder group 1 and (**b**) stakeholder group 2. Shaded bars represent those who provided scores in round 1 only; open bars represent those scoring in both rounds 1 and 2

Only one study to date has examined whether attrition bias is present between Delphi rounds in a COS project [55] although many ongoing COS studies are now planning to consider this. In the Harman et al. study (2015), average round-1 scores were calculated for each participant then plotted according to whether participants completed round 2 or not. Figure 4 provides two hypothetical scenarios representing the responses from two different stakeholder groups. In stakeholder group A, we can see that the average scores for those completing only round-1 (blue bars) are well contained within those average scores of those completing round 1 and round 2 (white bars). On average, participants staying in have scored outcomes similarly to those leaving the study, suggesting that attrition bias is unlikely to affect the results. For stakeholder group B, we can see that the average round-1 scores of those who did not complete round 2 are lower. If too many participants drop out of the Delphi process with lower previous round scores than the majority opinion, this will overestimate the level of importance of outcomes and over-inflate the degree of consensus.

Inevitably, examining average scores between completers and non-completers has its limitations. For example, non-completers may score some outcomes much higher than completers and score other outcomes much lower than completers, but average scores may remain similar between the two groups. Another approach to examine potential attrition bias would be to look at average scores of individual outcomes amongst those who do and not complete later rounds. If formally comparing average scores through statistical hypotheses tests it should be remembered that there will be an issue of multiple significance testing and false positive findings. However, such testing may enable identification of obvious patterns or differences in scoring between the non-completers and completers; for example, if non-completers are scoring patient-reported outcomes more highly than clinical outcomes but the reverse is seen for completers.

COS developers should consider the potential nature and cause of likely attrition bias when deciding how best to examine its presence. The assessment of attrition bias should be repeated for further rounds of the Delphi, that is, average round-2 scores should be compared for those completing round 3 and those dropping out after round 2.

##### Defining consensus

As for the scoring system, there are numerous ways proposed to define the consensus criteria, although the choice of criteria is rarely justified [136]; commonly these relate to a mean or median value for each outcome or a percentage of participants scoring an outcome as ‘important’. For example, Bennett et al. (2012) defined ‘consensus in’ (outcomes to be included in the COS) to be those for which 75% or more of participants scored 7 to 9 on a 9-point Likert scale [126], whilst others have suggested lower or higher rates scoring the categories deemed to be important. Some studies have also defined specific conditions or combination criteria as their consensus definition. For example, Schmitt et al. (2011) defined outcomes to be important if at least 60% of the participants scored 7 to 9 in at least three out of the four stakeholder groups being considered [127]. Wylde et al. (2015) implemented a threshold for inclusion in the core set of 70% of participants scoring outcomes as 7 to 9 and 15% or less scoring 1 to 3 to be met by both the clinician and patient panels or 90% or more scoring 7 to 9 from any single panel [137].

Williamson et al. (2012), describe the rationale for the ‘70/15%’ consensus definition that was in part used by Wylde et al. (2015); this approach has been used by others (Harman (2015) [55], Potter (2015) [56], Blazeby (2015) [58]) [21]. The idea is that the majority regard that the outcome should be in the COS, with only a small minority considering it to have little or no importance. It can similarly be argued that an outcome should not be included in the COS if the majority (70%) have scored an outcome of little importance, with only the small minority (fewer than 15%) consider it to be critically important.

The choice of what consensus criteria to use is an important consideration in COS development. Too accommodating criteria may result in a long list of outcomes that are not considered to be minimal whilst too stringent criteria can potentially exclude key outcomes that may otherwise have been included in the COS. Regardless of the consensus criteria that it used, it is important to define the consensus criteria in a protocol) to avoid any potential bias from changing the criteria after the Delphi results have been analysed [138].

##### Assessing the degree of consensus

**Fig. 5:**
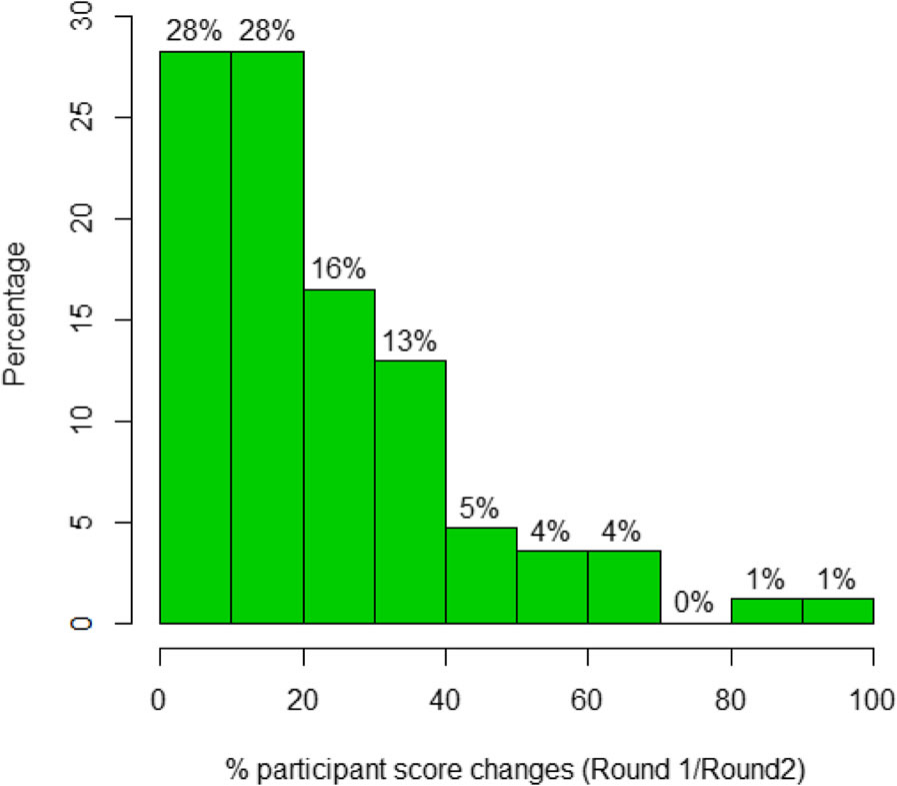
Percentage of scores changed between rounds 1 and 2 after viewing the results by stakeholder group

Some examination of the degree of consensus in each round is also advisable in order to ensure that the Delphi survey is working as a consensus method. As discussed earlier, the number of rounds in a Delphi can be dynamic but there will be a point beyond which a greater degree of consensus is unnecessary or unlikely to be achieved. One way to consider the degree of consensus is to examine the change in individuals’ scores between rounds. Brookes et al. (2015) [130] and Harman et al. (2015) [55] calculated the percentage of items for which a participant changed their score between rounds 1 and 2. Figure 5 presents the findings for the Harman et al. study. In this particular example, participants changed their opinions on only a small percentage of outcomes between rounds (indicated by the positive skew) which suggests that additional rounds would be unlikely to result in a much greater degree of consensus. Some ongoing COS studies are documenting the reasons for change between rounds [133, 134]; this may also help to determine whether additional rounds are useful.

One metric that may also be useful for examining improved consensus is the reduction in variability of individual outcome scores between two adjacent rounds. In their examination of the impact of different feedback methods, Brookes et al. [130] calculated the standard deviation of round 1 and round 2 scores for each item in the Delphi survey. Reductions in the spread of scores were seen between rounds 1 and 2. Reductions in interquartile ranges have similarly been examined in this way [139].

Whilst an examination of the degree of consensus is important to help validate the Delphi survey, it should be reiterated that the aim of the Delphi survey in the development of a COS is to determine which outcomes are core as opposed to achieving consensus for every outcome. Hence, it is unnecessary to conduct numerous rounds until consensus has been reached for all outcomes.

Finally, all methodological decisions should be fully reported and explained in the main publication. Any revisions to the original protocol should be documented with reasons. This will be covered in more detail in ‘Reporting guidance’ below.

##### 2.7.6.2 Face-to-face meeting

We recommend that representatives of key stakeholder groups have the opportunity for discussion of the results of the surveys to agree a final core set and undertake additional voting if required before a final COS is agreed.

Evidence for how such meetings are designed and conducted is lacking and published experience limited. A review identified just 10 examples of which nine included a face-to-face meeting [65]. Most held one meeting although a few studies undertook two or three. Some used a nominal group technique method to reach consensus although the majority had informal approaches. A nominal group technique allows all opinions to be considered initially, eliminates duplicate ideas and then asks participates to rank the importance of the remaining opinions [140]. This differs from traditional methods for decision-making which focus on the largest group initially supporting an idea.

Most meetings lasted about half a day, included some sort of voting and included presentations and discussion. Details of who moderated the meetings were often lacking.

##### Involving patients in consensus meetings

Another challenge in undertaking a COS study with patients as participants is that of enabling their inclusion in consensus meetings. This may, in part, be influenced by whether or not all stakeholders are brought together in a consensus meeting or whether these are run separately for patient participants. There are issues of power when multiple stakeholders work together to seek agreement. Spoken language and non-verbal communication in such meetings can exclude or subtly undermine patient participants. Some COS developers recommend that face-to-face consensus meetings are held separately for patients and professionals to allow patients’ views to be heard without contamination from other parties [56]. Other groups have brought patients and professionals together to discuss their views alongside evidence arising from a Delphi survey [55] and to make recommendations about a COS. With such mixed views, there is a need for research into this aspect of the consensus process.

Good facilitation is crucial, regardless of whether separate or combined consensus meetings are held. The preparation and support of patient participants both before and during the meeting is also vital. Consideration needs to be given to the specific needs of the patient group as they may have particular requirements to enable them to fully participate. The common principles in ensuring an accessible venue obviously apply, but there may be other considerations; for example, fatigue or pain from prolonged sitting need to be considered in planning such meetings. De Witt et al. (2013) provide information on barriers to participation for patients with rheumatological conditions in a face-to-face consensus meeting and make recommendations for facilitating their participation [141].

##### Other issues to consider

A crucial decision to be made is who to invite to the face-to-face meeting. Some COS developers have invited only the Steering Group involved in the project, which may or may not be those involved in an early Delphi study. Others have invited a combination of Steering Group members and other Delphi participants. An approach that may be helpful is to decide on the total number attending (which may be limited by resource and/or timing), and then to consider the desirable number from each stakeholder group. Some COS developers have aimed to include an equal split of health professionals and patients [133]. If Delphi participants are to be invited, some COS developers have included a final question in the survey about willingness to participate in a face-to-face meeting [133]. Participants for the meeting are then randomly selected from Delphi completers who noted that they were interested in attending. The advantage of this approach is that one can check that the final Delphi round scores are comparable for those attending and not attending which may give some reassurance that due consideration will be given to the evidence from the Delphi study.

Based on experience of consensus meeting facilitation, we recommend that the following further issues are considered: whether the facilitator needs relevant clinical experience or methodological experience, or whether co-facilitators may be appropriate; the independence of the facilitator; the ability of the facilitator to bring everyone in to the discussion without pressure; that the time needed for the meeting will depend on the aims and the number of outcomes to be considered; the structure for the meeting, and whether any small group or breakout group discussions will be held. We recommend that attendees are sent a reminder of their personal Delphi scoring prior to the meeting. The objectives of the project and the meeting should be made clear at the start, in terms of the scope of the COS, the emphasis on identifying core outcomes, and the methods to be used on the day. Thought should be given to the order of presentation of outcome results since it may be better to take the outcomes in groups/domains. Time should be allowed for at the end of the meeting to review the recommended list of outcomes holistically and agree the next steps which may include determining how each outcome should be defined and measured.

If a consensus meeting is to form part of the COS study, teams may need to consider how to address language issues in the meeting for non-native language speakers. If interpreters are to be used it is important to ensure that they are qualified to undertake that role, and meeting participants are reminded to speak in plain language to reduce the likelihood of difficulties with interpretation. A member of the team should brief the interpreters prior to the meeting, as to the purpose of the meeting, to discuss issues of confidentiality and to emphasise that information should not be filtered as this may bias responses. In planning the project, it is important to ensure that interpreting services are appropriately budgeted for. Interpretation issues are complex and we recommend that research teams seek advice from their own organisations as we have only provided a few basic considerations in this text.

### 2.8 Determining the core outcome set

The development of a COS may involve several components. Consideration should be given in advance to the criteria that will be used to determine when consensus has been achieved. Specification of the decision-making process to determine the final COS should be given in the study protocol which should reduce the risk that the people leading the process will define consensus post-hoc in a way that would bias the conclusions toward their own beliefs. For example, a study advisory committee (see Achieve global consensus’ below) may oversee the process whereby a review of the academic literature combined with stakeholder interviews informs a Delphi survey, the results of which are presented at a face-to-face meeting of representative stakeholders, and either ratified (in the case of an outcome meeting a pre-defined consensus definition for the Delphi survey) or further discussed and a decision made.

It is important to ensure that views from all key stakeholder groups are considered when making the final decision regarding the COS, and that the process for reaching that decision is reported transparently.

Researchers should consider the potential impact of the following methodological decisions on the final results: group composition, questioning technique, the information that participants receive to inform their answers, whether or not responses are anonymous, how the group participants interacted with, or influenced, each other, the medium of the interaction, attrition bias, analysis which can miss or overstate the importance of certain outcomes, and the way in which consensus is reached.

All of the above require further investigation to develop transparent, reproducible and robust methods for decision-making during the COS development process. Until this is available it is recommended that reporting details of the process undertaken follows the recommended guidelines [142].

### 2.9 Qualitative methods in core outcome set development

As indicated throughout this handbook, COS development can involve several different stages as well as a mix of both research and consensus processes. Some COS developers have recently started to use qualitative methods as part of the wider COS development process [55, 74, 89, 133, 143, 144]. In this context qualitative methods may be useful for accessing the perspectives of groups, such as patients, carers, members of the public and health professionals, whose views may not be encompassed in systematic review of outcomes. Making COS development meaningful for these groups can be challenging and, as we outline below, qualitative methods can help COS developers to navigate this challenge. However, it is important to note that much remains to be learnt about the use of qualitative research in COS development and this section is based on experience of a limited number of projects.

#### 2.9.1 Why use qualitative methods in core outcome set development?

##### 2.9.1.1 To identify outcomes of relevance to the whole community of stakeholders

COS developers may conduct systematic reviews of the outcomes measured in published studies to develop ‘long lists’ of outcomes to go forward to consensus processes such as a Delphi survey. However, the opinions of clinical trial designers and researchers will inevitably have influenced the outcomes used in published studies and these same opinions will also be reflected in the findings of systematic reviews. Patients, carers and the public have historically had little say in what outcomes are measured in studies, so systematic reviews can overlook important outcomes. For example, the OMERACT group have pointed to how fatigue – an outcome of crucial importance to patients – was overlooked in rheumatology trials until relatively recently [28]. Qualitative studies with patients and other stakeholders can help to ensure that the long lists of outcomes that go forward to a consensus process are comprehensive from the perspective of the whole community of relevant stakeholders, not just the groups that have historically influenced what outcomes are measured in research.

##### 2.9.1.2 To preserve the distinctive perspective of different stakeholders

Most people do not naturally think or talk about their experiences of health conditions, illness and treatments within an outcomes or research frame of reference [145]. In order for patients and other stakeholders to participate meaningfully in consensus processes, such as Delphi surveys, COS teams need to help them to understand what outcomes are, how these are used in trials and why COS are needed. This involves patients learning things about research which, by definition, involves influencing them. This learning could also potentially diminish the distinctiveness of their perspectives as patients. Qualitative studies can enable patients and other stakeholders to participate in COS development in ways that minimise such influences. For example, qualitative interviews involve asking participants open questions and allowing them to respond in their own words. Rather than asking patients to learn about outcomes or why COS are needed, the researcher adapts to the patients’ world and works within their existing capabilities. The overall purpose of the qualitative research must of course be clearly explained, but with a well-thought out qualitative study design and prompt guide (interview schedule) patients can describe their experiences of illness and treatment in ways that are both intrinsically meaningful to them and which simultaneously help the COS developer to identify what outcomes are important to patients.

##### 2.9.1.3 To help make consensus processes accessible to patients

When patients and carers come to rate or vote on lists of outcomes during consensus processes, the outcomes need to make sense to them. Because qualitative research involves patients describing their views and experiences in their own terms, it gives researchers insight into how patients naturally conceptualise outcomes and the language they use. COS developers can use the findings from interviews and focus groups to make subsequent consensus processes accessible to patients. This might be by using the qualitative findings to take account of the patient perspective in deciding on the scope of a COS, and to ensure that the labelling and explanation of outcomes is understandable to patients.

##### 2.9.1.4 To inform deliberations in the final stages of core outcome set development

Qualitative study findings can also illuminate *why* outcomes are important to patients, which may usefully inform the final stages of COS development if there is divergence between stakeholders about which outcomes are core and which are not.

##### 2.9.1.5 To address gaps in existing core outcome sets

Where an existing COS has been developed without the perspective of patients or other key stakeholders, qualitative studies may help to address this omission. This would usually be as part of a wider review process as described in Chapter 3.

#### 2.9.2 In what circumstances might core outcome set developers consider using qualitative methods?

Whilst qualitative methods can be helpful for the reasons described above, we recommend that they are usually used as part of wider COS development process. Qualitative studies are not designed to include large representative samples and so these designs are not suitable for estimating how many patients think that a particular outcome is important. Moreover, qualitative research studies are not consensus processes and analysis of interview or focus group data leaves considerable room for interpretation. Whilst this analysis can be documented and public research partners and other team members can be involved, to achieve a final COS that users will have confidence in COS developers will need to use processes that have been specially designed to reach consensus.

In deciding whether to incorporate qualitative methods as part of a wider COS development process, developers should consider the following:

- The specific purpose for which qualitative evidence is needed.
  Whilst qualitative methods can be used to access the perspectives of patients, carers and other stakeholders [133, 146] developers will find it helpful to specifically consider what it is that they are hoping to achieve through the qualitative work, and whether a qualitative approach is the most suitable way to achieve these aims. For example:
  - о A COS developer may be concerned that not all potentially important outcomes have been identified to go forward to a consensus process and will use qualitative work to help ensure that no potentially important outcomes are missed
  - о Retention of Delphi participants over several rounds can be problematic and qualitative studies may help to minimise the number of Delphi rounds needed. For example, qualitative research may enable developers to omit the initial ‘blank page’ or open-ended round of a Delphi survey
  - о COS developers may be working with stakeholder groups, such as patients with dementia or learning disabilities, for whom other COS development activities are unsuitable
  - о As we note above, qualitative research findings may also help developers to define the scope of a COS by informing the choice of population and interventions to be covered by a particular COS
  - о As also noted above, qualitative research findings may help to ensure the consensus process (language, explanations, etc.) is accessible to patients
  - о COS developers may need insights on why particular outcomes are important to patients; qualitative research is widely regarded as being useful in addressing ‘why’ questions
- Whether existing qualitative studies have been conducted that could address some of these aims.
  Many qualitative studies have now been published describing patients’ and carers’ experiences of specific conditions and treatments. It may be possible to use this existing work to identify how well outcomes from systematic reviews of trials map to those of patients. Similarly, qualitative datasets may be available for secondary qualitative analysis that could serve the same aims. If previous qualitative evidence or data are available, COS developers will want to be clear about why additional primary qualitative research is needed.
- Qualitative research requires specialist methodological expertise and COS developers will need to ensure that their team includes this expertise. Similarly, the collection and analysis of interview or focus group data requires time and resource and COS development teams will also need sufficient funds to support the qualitative work.

#### 2.9.3 Issues to consider in designing primary qualitative research to inform core outcome set consensus processes

It is beyond the scope of this section to provide guidance on how to do qualitative research. This is already covered by an extensive literature. However, there are some specific issues in conducting qualitative research within COS development that may be particularly relevant for COS developers. Before turning to these we would like to emphasise that the issues we identify below are not exhaustive. We hope that COS developers will reflect critically on our suggestions, rather than regarding them prescriptively.

##### 2.9.3.1 How the qualitative research and patient involvement complement one another

To an extent qualitative research and the involvement of public research partners may share similar overall goals. For example, both may aim to optimise the accessibility of the language used in the consensus process. Nevertheless, the contributions of public research partners, though crucial in COS development, cannot substitute for qualitative research.

##### 2.9.3.2 Sampling

COS developers will usually aim to access participants who have direct experience of the illness, treatment or care process relevant to the COS as patients or service users. Additionally, the perspectives of carers, parents or professionals may also be valuable, for example where the capacity of patients to articulate their experiences is limited or where carers’ perspectives on outcomes are of interest in their own right. Sampling to qualitative studies is usually purposive (e.g. aiming for maximum diversity), rather than probabilistic (aiming for statistical representativeness [147]), but it should be noted that purposive sampling is not the same as convenience sampling. Whilst patient organisations or charities might offer a convenient route for accessing stakeholders, individuals who are contactable via such organisations may differ in important ways from the wider community of patients. If COS are to reflect the perspectives of this wider community, developers will want to access patients across a spectrum of sociodemographic and other characteristics. Sampling via different clinical, health and community settings will likely be best suited to this.

##### 2.9.3.3 Eliciting participants’ perspectives

We have noted above that qualitative research may be particularly useful in COS development because it enables participants to talk about their priorities in their own terms. Even without an overt focus on outcomes or COS [148] – a frame of reference which may confuse patients and shape or colour what they say – much can be learnt from patients’ naturalistic accounts of their experiences and perspectives. A narrative style of interviewing can be helpful here, particularly at the outset of an interview. Beyond this, the questions and prompts can be more overtly focussed on outcomes although questions still need to be tailored to the participant group and topic, and be responsive to individual participants. As well as examining the qualitative academic literature relevant to COS development, in designing the interviews and prompt guides qualitative researchers should consult with patient research partners [149]. We offer the following further suggestions as tentative pointers and hope that COS developers will treat them only as starting points for developing their own questions and prompts:

- Interviews might begin by asking participants to talk about their actual experiences, including how the illness and treatment has affected their lives, about treatment decisions, how these decisions were made and what influenced them.
- Over the course of an interview, questions may become more focussed on outcomes and opinions; for example, asking patients what they had hoped for from their previous treatments or what they would want from a new treatment if one became available. Understandably, many patients with chronic conditions might hope for curative treatments and interviewers may need to be prepared to prompt patients to describe expectations that are more immediately achievable.
- Towards the end of interviewing, questions might become more research- or COS-specific. For example, the interviewer might summarise outcomes that patients have discussed in previous interviews and explore what words or phrases patients think researchers should use to label or explain outcomes for future patients.
- Where the qualitative work has a very specific focus; for example, informing an online Delphi survey for a particular stakeholder group, interviewing might focus on a prototype of the survey and use think-aloud techniques [103, 104] to explore how stakeholders interpret the outcomes and how the phrasing might be refined.

##### 2.9.3.4 Data analysis

COS developers have drawn on a range of analytical orientations or approaches to qualitative analysis such as framework analysis [150], constant comparative method [146] thematic analysis, as well as interpretive phenomenological analysis [151]. Qualitative researchers will be best placed to identify an approach that best suits their aims. If interviews have been broad ranging or narrative, from an early point it will be important yet challenging to focus the analysis on those aims that are most pressing. For example, identifying what outcomes to go forward to the consensus process and how these are labelled may need to take precedence over identifying why certain outcomes are important to patients. As for any qualitative analysis, interpretation will need to contextualise the data and not just catalogue data extracts. This will mean considering what things patients might be reluctant to speak about as well as what might be taken for granted in the context of certain illnesses or treatments. In interviews stakeholders may not directly articulate some outcomes and identifying these may call for considerable interpretive work. For example, it took considerable qualitative work to identify empowerment as an outcome of genetic counselling [152, 153]. Finally, whilst qualitative researchers are likely to lead the analysis, they will want to closely involve other members of the COS development team, including the public research partners, to ensure the analysis is informed by a range of perspectives.

##### 2.9.3.5 Writing up the findings

Guidance is available on writing up qualitative research [154], so here we focus on those aspects that may warrant particular consideration by COS developers. It should be clear what has been discovered from the qualitative work (i.e. how the findings add to what was previously known) and how the qualitative work has contributed to the COS development process. This might include a commentary on whether the qualitative work has identified potentially important outcomes beyond those already identified in systematic reviews of trial outcomes, how the qualitative work has informed the scope of the COS or how it has informed the language used in consensus process. COS developers will need to decide whether to publish the qualitative research separately from the other elements of the COS development process, or combine all elements in one article. Where the qualitative research is combined with other elements of the COS development process, authors may find it helpful to consult articles on writing up mixed-methods research [155]. Where the qualitative findings are published separately, it should be clear that the qualitative study is linked to the wider COS development process so that subsequent COS developers can learn from this work and unnecessary duplication can be avoided.

### 2.10 Considerations to enhance patient participation in a core outcome set

There are numerous challenges in facilitating patient participation in a COS study and these will depend on the patient group and the methods chosen. In the following discussion we explore some key challenges for patient participation in consensus processes such as Delphi studies. Patient participation through qualitative methods was discussed in the previous section. A checklist to support COS developers working with patient research partners in designing COS studies is available on the COMET website and may help with planning to address some of these challenges at the outset.

#### 2.10.1 Accessing patients

Involving patients as participants in COS will require consideration of the need for ethical approval in each country where the study is taking place. The current situation in the UK is that ethical permission is required if the goal of COS development is to produce generalisable knowledge. Guidance regarding the need for ethical approval has been developed by the COMET PoPPIE (People and Patient Participation, Involvement and Engagement) Group [156].

Like any study with this aim, accessing patients or other groups to participate in a COS study requires consideration of sampling strategies as appropriate to the research method being used. Patients may be sampled from primary, secondary and/or tertiary health care settings depending on the condition under study. The approaches for accessing patient participants may depend on whether participants are being sought for an online or face-to-face Delphi survey. Methods for accessing patients may also depend on whether the patient participants are being asked to join a consensus meeting with other stakeholders, where COS developers may specifically decide to select patients who can take on advocacy roles in this context and so help to ensure that the patient perspective gets heard.

Patient organisations may provide a route to access patients for certain conditions but their members may have special interests and may differ from the wider patient population (in terms of age, gender, socioeconomic status, ethnicity and other relevant characteristics). If patient organisations are to be used, purposive sampling techniques may help to ensure that a diverse sample is accessed. Patient communities also exist in social media, but COS developers need to be cautious about using them. There is evidence of poor response rates through social media and those who do respond may include a limited range of the patient population due to self-selection [157]. Links to guidance on the use of social media can be found on the COMET website. COS developers also need to ensure that patients or patient advocates from patient organisations have relevant experience of the condition and that this experience is relatively recent. Further discussions about sampling of patients to take part in a COS can be found in the previous section.

In terms of promoting the COS study to potential participants, COS developers might consider a range of sources; for example, clinic waiting areas and through patient organisations and they might also plan for how to promote the study within hard-to-reach communities. Patient organisations and public research partners can help to advise on where studies might best be promoted for patients with a particular condition.

#### 2.10.2 Information for patients

The way that COS studies are explained, finding the right language to do so and asking questions about outcomes with a range of stakeholders are other key challenges. COMET has developed two plain language documents with the involvement of patients: one explains what COS are and what the COMET Initiative is; the other describes what a Delphi study is. These resources can be found on the COMET website [109] and may be useful in developing information for COS studies.

When communicating with patients and when developing written study information and questions about outcomes, it is important to use plain language. Free resources to help with writing in plain language are available online. Specific guidance on writing for people with particular needs, such as adults with learning disabilities, might also be sought where relevant, e.g. easy-read publications. Readability tools are also available to provide an indication of how readable study material is. For further information on relevant resources to help with writing and assessing readability see the COMET website.

Considering how to present written information can help with ensuring its accessibility. Any specific visual needs, such as colour blindness or sight problems, also need considering when designing participant materials.

For certain populations and if funding allows, it may be appropriate to provide information about the study through other media; for example, podcasts or video presentations. Consulting with patients and patient organisations in the design stage of the study will help to identify the most accessible means of providing information and ensuring its acceptability to the relevant groups.

#### 2.10.3 What questions to ask when involving patients as participants in a core outcome set study?

Asking the overarching question about which outcomes are relevant and important to patients can be difficult as the word ‘outcomes’ may not commonly be understood. Providing examples of outcomes in conditions can be useful, but care needs to be taken in selecting an example that will not bias respondents. In a systematic review of COS, Gargon et al. (2014) found that the precise question used to ask about outcomes was not always reported [14]. It is important that we report on such aspects of study design so that we can develop best ways of engaging patients as participants in future COS studies.

Patient involvement is important in developing questions for use in a COS study. Examples that we know about are from the ACORN and the MoMENT studies. The Acne Core Outcome Research Network (ACORN) consulted with patients and clinicians when developing their question for a COS study on acne. They showed patients several questions and asked which would be the most appropriate to use in a survey to include patients. Following this consultation they decided on the question: ‘Please tell us in your own words how you decide if your treatment has been effective. Physicians do things like counting spots or using an improvement scale but how do you, or will you, do it?’ This question was incorporated into a James Lind Alliance Priority Setting Partnership international survey about research priorities for acne [158]. The MoMENT study (described below) developed their question through their study group with the involvement of the chief executive from a patient organisation and then piloted their question with patients.

Further examples of how researchers have previously asked patients/carers about outcomes of importance are presented below. Some questions focus on treatment and others focus on the patient experience of a condition [89]. The scope of the COS may influence the type of questions used; for example, particular question types might work best for COS where the scope relates to a particular type of intervention, whilst other studies may work better with more generic experiential questions:

- The MoMENT study [55] – this was a study to develop a COS for the management of otitis media with effusion in children with cleft palate. A consensus survey was used in this study. Parents were presented with a list of outcomes to score but could add in any missing outcomes that they considered relevant. The question used was: ‘Think about when your child had glue ear and how you might decide if their treatment for glue ear had worked. We would like you to look at the list below and tell us how important each thing on this list is in deciding if treatment has worked’ [55].
- An online Delphi consensus study was conducted with patients who suffered from migraine. The patients were asked two open-ended questions in round 1 of the Delphi survey and these were:
  - о What do you find most bothersome about having a migraine attack?
  - о If a new medicine was developed against migraine attacks, what would you wish the effect of this medication to be [159]
- A COS was developed for children with asthma [60]. In an online Delphi survey, parents, young people and clinicians were asked open-ended questions in round 1 to identify outcomes of importance. The following four open-ended questions were used with the parents and young people:
  - о Over the last 12 months, have you generally felt that the regular preventer treatment that your child (you) takes has kept their asthma under control? Yes/No. If you ticked ‘Yes’, please tell us what aspects of your child’s (your) asthma, or their daily life, have made you feel happy that they are on the correct regular medication. If you ticked ‘No’, please leave this question blank
  - о Over the last 12 months, have there been times when you felt that your child’s (your) regular preventer treatment should be increased or changed because their (your) asthma was not under control? Yes/No. If you ticked ‘Yes’, please tell us the reasons why you were not satisfied with the regular preventer treatment that they (you) were taking? If you ticked ‘No’, please leave this space blank
  - о Does anything worry you about the fact that your child (you) has asthma? Yes/No. If you ticked ‘Yes’, please tell us the worries you have about the fact your child (you) has asthma. If you ticked ‘No’, please leave this space blank
  - о Does anything worry you about the regular preventer treatment that your child (you) takes for their asthma? Yes/No. If you ticked ‘Yes’, please tell us what worries you have about the treatment your child takes for their asthma. Please be as specific as you can. If you ticked ‘No’, please leave this question blank

#### 2.10.4 Maintaining patient involvement throughout a consensus process

Consensus processes, such as Delphi surveys, may run over several months and strategies to maintain the involvement of patients in this process are important. Involving patients and patient organisations in designing and overseeing the COS may help COS developers to plan for this at the outset. Regular communication with participants in COS may be important in maintaining their engagement – for example, updates on the progress of the study and forewarning of anticipated dates of subsequent survey rounds. Some researchers have considered incentives; for example, prize draws, as a mechanism for maintaining participation. Ethical guidance will need to be sought for this and patients involved in designing your COS study can help developers to identify the most appropriate incentives for particular patient groups; for example, young people may prefer a different incentive than those of other generations.

#### 2.10.5 Disseminating survey results to patients/the patient population

Having taken the time to be involved in a COS study, patients should be offered access to the survey results in an accessible way and the results may also be of interest to the wider patient population; for example, through patient organisation newsletters. The rules of plain English again apply (see above) in developing such end-of-study information and patient research partners can help teams design accessible end-of-study information. Morris et al. (2015) involved public research partners throughout their research and in writing the end-of-study information for a ‘children with neuro-disability’ study (which included a COS) [79]. Links to this end-of-study information can be found on the COMET website.

### 2.11 Determining ‘how’ to define and measure an outcome in the core outcome set

The text above discusses the approach to establishing consensus about what outcomes are important to measure. Consensus is also needed on how selected outcomes should be defined and measured. Of 227 published COS, 84 (37%) considered both the what and the how to measure in the same study [14, 39]. A review of methods used in these studies to determine how to measure the chosen outcomes is currently underway.

Different outcomes may be measured by a single question, a questionnaire, a performance-based test, a physical examination, a laboratory measurement, an imaging technique, and so forth. A variety of either definitions, measurement instruments or devices is often found to be used for the same outcome. For example, in a review of outcomes for colorectal cancer surgery some 17 different definitions were identified for ‘anastomotic leakage’ [85]. In a review of PROMs from studies evaluating radical treatment for oesophageal cancer, searches identified 21 generic and disease-specific PROMs containing 116 scales and 32 single items with 94 different verbatim names [86].

#### 2.11.1 Choice of measurement instrument for an outcome

Evidence synthesis is further hampered by incomparable scores from different instruments and variability in the quality (reliability and validity) of measures used [35]. Guidance is needed on how to select the best instrument for a given outcome.

A joint initiative between COMET and COSMIN aimed to address this gap by developing a guideline on *how* to select outcome measurement instruments for outcomes included in a COS [160].

Based on a Delphi study amongst a panel of international experts, consensus was reached on four main steps in the selection of outcome measurement instruments for a COS:

- Step 1. Conceptual considerations
- Step 2. Finding existing outcome measurement instruments, by means of a systematic review and/or a literature search
- Step 3. Quality assessment of outcome measurement instruments, by means of the evaluation of the measurement properties and feasibility aspects of outcome measurement instruments
- Step 4. Generic recommendations on the selection of outcome measurement instruments for outcomes included in a COS

This consensus-based guideline can be used in defining *how* to measure core outcomes for any disease or condition in health and social care [44].

It may be that several measurement scales exist for measuring a particular outcome, such that steps 2 and 3 may take some time. As an example, fatigue was identified by patients with rheumatoid arthritis to be an important outcome to be included in the COS. A systematic search for articles measuring fatigue discovered 23 scales. Applying the OMERACT Filter for truth, discrimination and feasibility, six were found to have sufficient evidence of validity to pass most criteria [30]. In 2006, fatigue was endorsed as an additional core outcome at the OMERACT 8 meeting following further work, undertaken to demonstrate responsiveness [30].

In general, it is recommended that once all definitions, tests, questionnaires, techniques, etc. for measuring a particular outcome have been identified, and their properties assessed, a further consensus process should be undertaken to agree how each should be measured.

### 2.12 Achieve global consensus

To compare and contrast all research in a topic area, a COS must be applicable and adopted across relevant settings and disciplines including internationally where appropriate.

Of the 227 COS studies that were identified in the systematic review, the majority have involved collaborators (*n* = 180, 79%) and participants (*n* = 154, 68%) from Europe and/or North America. In contrast, the remaining continents have been involved as collaborators in just over one fifth of studies (*n* = 47; 21%) and have participated in less than one third of studies (*n* = 73; 32%). The geographical locations of collaborators and participants who have been involved in developing COS are presented in Table 4.

**Table 4:** Geographical locations of collaborators and participants involved in core outcome set (COS) development

	Collaborators *n* (%)	Participants *n* (%)
North America, Europe	78 (34)	62 (27)
North America	53 (23)	50 (22)
Europe	49 (22)	42 (19)
North America, Europe, Australasia	13 (6)	15 (7)
North America, Europe, Asia	6 (3)	12 (5)
North America, Europe, Australasia, Asia	6 (3)	10 (4)
North America, Europe, Australasia, South America, Asia	4 (2)	10 (4)
Australasia	3 (1)	2 (1)
North America, Australasia	3 (1)	2 (1)
Europe, Australasia	3 (1)	1 (<1)
North America, Europe, South America, Asia	2 (1)	3 (1)
North America, Europe, Australasia, South America	2 (1)	1 (<1)
North America, Europe, Australasia, Africa	1 (<1)	3 (1)
North America, Europe, South America	1 (<1)	3 (1)
North America, Europe, Africa	1 (<1)	1 (<1)
North America, Europe, South America, Africa	1 (<1)	1 (<1)
Asia	1 (<1)	
North America, Europe, Australasia, South America, Asia, Africa		4 (2)
North America, Europe, Australasia, South America, Africa		2 (1)
North America, South America, Asia, Africa		1 (<1)
North America, Europe, Australasia, Asia, Africa		1 (<1)
North America, Europe, South America, Asia, Africa		1 (<1)

Health professionals from multiple countries have been engaged through both professional societies [59] and personal networks [161, 162]. The inclusion of patients from multiple countries is likely to be more difficult. Some groups have included patients from different countries but usually in small numbers based on personal contact [161]. A novel approach involves health professionals interviewing several patients in multiple countries following training and according to a common protocol [163].

Interviews with COS developers have highlighted some of the considerations of undertaking this work internationally [65]. A prominent question amongst COS developers interviewed was whether a COS *should* be developed internationally. This links to the intended reach of the COS recommendations. If the COS is intended to be used globally then this has implications for how COS are developed, who is involved in that process and the resources required. There was no consensus amongst those in the study as to whether COS should be developed internationally or not.

Both published and ongoing developers talked about the challenges of undertaking COS development internationally, particularly the linguistic challenges that global participation entailed and the need to translate concepts and questionnaires. They spoke of the logistical and resource challenges of organising an international meeting, and the challenge of getting the balance between what is ideal and what is pragmatic. International ethical approval procedures were described as resource intensive and ‘bureaucratic hurdles’.

Considerations about efficiency and heterogeneity arise with global development, as well as generalisability. However, heterogeneity can be as great within countries as between so this should not serve as a barrier to internationally developed COS. In a letter to an editor in reference to the international HOME COS for eczema, it was pointed out that for a disease with global impact there was limited representation of non-western participants from countries where the societal burden of the disease is high [164]. If a COS is developed to have international applicability then there is an issue of inclusivity that needs to be addressed. The question of international representation in COS development is one that requires further research.

For practical and resource reasons, such as those described, stakeholders from a limited number of geographical areas may have been involved in the development of a COS. Consideration should be given to the generalisability of the results and the need for any further research involving additional stakeholders if the COS is to be used in settings other than the one in which it was developed.

### 2.13 Study team and study committees

There are a number of study committees, described below, that can be helpful in the development of a COS.

#### 2.13.1 Study Management Group

The Study Management Group (SMG) is responsible for the day-to-day management of the study and should meet regularly (usually monthly but frequency may vary depending on study activities).

The SMG should represent a multidisciplinary skill set relevant to the study. For example:

- A clinical lead in the area covered by the COS
- A study coordinator with understanding, and ideally experience of systematic review and/or outcomes and/or Delphi studies
- A qualitative researcher – if undertaking qualitative components of the study and not covered by the experience of the study coordinator
- PPI research partners – ideally a minimum of two to provide support and cross cover
- Other clinical or methodological experts depending on the individual needs of the study

#### 2.13.2 Study Advisory Group (SAG)

Where a COS is developed for a condition involving a large multidisciplinary health care team, representation of all disciplines on the SMG could make that group unmanageable. Instead a SAG could provide additional expertise for each discipline.

The timing and frequency of SAG meetings should be considered based on planned study activities. Generally, the SAG would meet less frequently than the SMG with meetings scheduled at critical points in the study that require multidisciplinary input. Such tasks may include review of the categorisation and description of outcomes, decisions regarding the structure and content of the list of items to be considered in a consensus process, and review of the final report following the consensus meeting.

#### 2.13.3 Costing the project

There are a number of cost areas to consider. The list below describes some of the key costs but this is not exhaustive and each study will have particular considerations. The costs of a study will likely vary over the study duration, and a study GANTT chart that maps out when key activities will take place is a useful tool to help estimate the resources needed at each stage of the study.

##### 2.13.3.1 Staff

The staff working on a study will vary depending on study complexity and should be considered on a case-by-case basis. Staff involvement may also vary over the duration of the time depending on planned activities.

Staff roles to consider:

- *Co-applicants/members of the Study Management Group* – who are the co-applicants of the study, what role will each have and how much time will they need to fulfil that role including contributions to the final report and study dissemination?
- *Statistical support* – consider statistical support for study design, analysis and reporting
- *Information systems* – will a study database or bespoke software be required? If so include time for an information systems developer to provide these resources
- *Data management* – consider any data management requirements of the study. For example, if using paper-based questionnaires, who will enter responses into the study database and who will populate and distribute follow-up questionnaires, if appropriate?
- *Study coordination* – the amount of coordination will depend on the activities of an individual study. Generally, study coordination time to schedule study meetings, distribute and follow-up survey responses and to schedule and prepare documentation for focus groups, consensus meetings, etc. should be considered. The coordinator may also take responsibility for systematic reviews and other methodological areas of the study depending on experience, financial management and communication with the funder, ethical or governance approvals needed for the study, monitoring of participant consent, etc. and preparation of the final study report
- *Qualitative research* – consider any qualitative aspects of the study and include time for data collection, analysis and write up

##### 2.13.3.2 Software

The software chosen will depend on the needs of the study. Basic online survey software may be available at an institution level or might require an annual licence to be purchased

If in-house software is being developed then the time required by information systems developers, together with the cost of server space, should be considered (see ‘Staff’)

Delivering a Delphi survey on-line may benefit from a bespoke system that allows automated reminders and feedback of results. The cost of developing a bespoke system should be considered against the staff costs where manual population of data for each round and reminders are needed.

##### 2.13.3.3 Websites

If the study and/or survey will be hosted on a study-specific website then the cost of domain name registration and annual server hosting costs should be included. These costs are usually on an annual basis and cannot be included pro-rata (particularly domain name registration).

##### 2.13.3.4 Printing

In some circumstances information may need to be provided in hard copy. Examples include Participant Information Sheets, Consent Forms, postal surveys or Delphi questionnaires, study summaries, etc. If only a small number of participants are expected then costs might be included in a general consumables budget. However, where large numbers are needed these should be included separately. Where printing costs are included separately these should include an initial print run plus at least one amendment of materials.

##### 2.13.3.5 Meetings

Meetings should be included where the meeting incurs costs. Face-to-face meetings should consider the cost of meeting rooms, refreshments and travel. Teleconference costs should consider the number of lines needed and the location of those joining (national or international). University or hospital telecoms may be able to provide teleconferencing facilities and pricing structures.

Types of meetings to consider are:

- Study Management Group and Study Advisory Group (SAG) meetings (viii) – consider frequency, whether the meeting is face to face requiring travel costs or by teleconference only. Take into account the location of participants, local, national, international
- Consensus meetings – you may need to include the cost of venue hire and subsistence; some locations will provide a per person cost that includes all facilities and subsistence. The location of the meeting and subsequent travel costs for participants should be included. If national and/or international participants are attending a location close to a main train station or airport would be most appropriate and the cost of venue hire can be considered against additional travel costs for attending an institutional venue
- Focus groups – take into account who the participants are. If health care professionals then consider where the meeting will take place, travel costs and subsistence. Focus groups for patients/parents should also include travel and subsistence for each person. Focus groups for patients/parents might take place in an alternative setting that also acts as an incentive for attendance. For example, holding the session at a zoo or an aquarium which allows participants to access the attraction free of charge after the meeting. Childcare/carer needs should be included for patient (see ‘PPI’ section below for cost calculator)

##### 2.13.3.6 Travel

Travel for meetings has already been described. Applicants applying for funding should review their institutional policies on travel; these may include the purchase of standard class fares only and a limit on the mileage that can be incurred for a single trip without justification.

##### 2.13.3.7 Transcription

Transcription costs for interviews, recorded meetings or focus groups may need to be included. Each institution will have a list of approved suppliers and quotations should be sought prior to applying for funding. Prices are usually provided per minute and will increase where there are multiple speakers who need to be identified in the transcript.

##### 2.13.3.8 Translation

Translation costs should be included for each language appropriate to the trial. Translation would usually include forward and back translation of documents to ensure consistency between languages. Again institutions will usually have a list of approved translation services who can be approached to provide a cost per document.

##### 2.13.3.9 Patient and Public Involvement (PPI) in the design and conduct of a study

In some countries, e.g. UK, payments are offered to public research partners for the time involved in undertaking their PPI activities. The acceptability of and approaches to payment for PPI may differ by country, hence advice should be sought locally. If patients are paid for PPI activities, researchers need to ensure that patients are aware of any implications of being paid for involvement, e.g. impact on any benefits that they are entitled to or tax implications.

Costings should be estimated for each type of involvement. For example:

- PPI membership of study committees/groups
- PPI co-applicants
- Other PPI activities. Examples include parent/patient facilitators of workshops or consensus meetings
- Training – consider whether PPI contributors require any training and who will provide this. This could include attendance at conferences, external training events or internal training. The time to attend the training together with travel, subsistence and childcare/carer costs should be included
- Childcare/carer time

Advice on costs for PPI in the UK is available via the INVOLVE website [165]. This site also includes resources for estimating PPI costs [166].

##### 2.13.3.10 Equipment

Software and equipment is available to allow anonymous voting at face-to-face meetings. It might be possible to borrow this from an institution or it may need to be purchased.

If focus groups or consensus meetings need to be recorded then you may need to consider purchasing equipment to do this or to employ external companies with professional equipment, particularly if it is a large group.

Also consider laptops/PCs that are needed for study staff, particularly full-time members of staff employed specifically for the study.

##### 2.13.3.11 Incentives

You may like to offer an incentive to study participants; this might be acknowledgment in the publications, a gift voucher for completing an interview or joining a focus group or a prize draw for those completing a survey. Examples of funded incentives include an entry into a prize draw for an iPad mini for those completing an online Delphi survey (one iPad mini for each stakeholder group, i.e. health care professionals and parents), a £20 gift voucher for parents attending a focus group, a £10 gift voucher for parents completing a qualitative interview [55].

##### 2.13.3.12 Systematic review

If completing a systematic review as part of the study, costs, excluding staff costs, may include printing and costs for unsubscribed journals. Each institution should be able to provide a cost for an interlibrary loan and an estimated number of these included based on the estimated size of the academic literature, e.g. estimate approximately 10% of included papers.

##### 2.13.3.13 Publication

Costs to publish the protocol and the final manuscript should be included, based on the current costs for the desired journal.

### 2.14 Reporting guidance

COS developers should provide a clear and transparent report of the methods they used. Reporting standards for a Delphi survey component of a COS study have been proposed previously by some authors of this Handbook [138]. A more general checklist of items to be reported for a COS study was then published [21].

The first comprehensive systematic review of COS highlighted the need for a more formal reporting guideline for COS development studies due to the amount of relevant information that is missing from journal articles [14]. A guideline for reporting COS studies has recently been developed [134].

### 2.15 Quality assessment/critical appraisal

It has previously been suggested that the potential impact of the following methodological decisions on the final results should be considered [21]: group composition, questioning technique, the information participants receive to inform their answers, whether or not responses are anonymous, how the group participants interacted with, or influenced, each other, the medium of the interaction, attrition bias, analysis which can miss or overstate the importance of certain outcomes, and the way in which consensus is reached.

However the definition of quality is a difficult one, and an area for further research may be to achieve consensus regarding minimum standards, as discussed in Chapter 4.

## Chapter 3: Implementation, review and feedback

### 3.1 Background

Those developing a core outcome set (COS) need to give careful consideration to how it will be used. This will help to ensure that the COS contributes to the reduction of research waste, without the COS development itself being wasteful [167]. Those developing the COS might prepare an implementation plan as part of the development process which might include both the intended audience and users of the COS and the pathways to reach them. In this chapter, we consider efforts to facilitate the implementation of COS, including what potential users might consider when deciding whether a COS is appropriate for their purposes. We also note how research might be embedded into the implementation activities to help build the evidence base for this important aspect of COS development, bearing in mind that COS development is still in its relative infancy and implementation strategies have not been a prominent feature in their development to date. The chapter also considers how COS might be kept under review, to help with decisions about revising them.

The first section of the chapter describes examples of existing research on the uptake of COS, highlighting how the rarity of such studies means that there are many uncertainties about the impact of the hundreds of COS that have been developed to date and about the most appropriate methods for the implementation of COS. This is followed by a discussion of the role of various actors in the implementation of COS, including the COMET Initiative, COS developers, researchers, such as trialists and systematic reviewers, funding bodies and journal editors. We then move on to discuss the importance of keeping COS under review to ensure that COS remain valid and up-to-date; before concluding with suggestions for future research into the uptake, implementation and maintenance of COS.

### 3.2 Existing research on the uptake of core outcome sets

The rarity of research into the uptake of COS is not surprising given the relatively small number of COS. For comparison, researchers into the impact of randomised trials would be able to draw on several hundred thousand trials and those investigating the impact of systematic reviews have many tens of thousands of examples of this type of research as their starting point [168]. In contrast, fewer than 200 COS have been in the public domain for more than 5 years [14, 39], and there is likely to be a lag between publication of a COS and its appearance in trial registries, trial reports and systematic reviews due to the time needed to design a trial, obtain funding, and subsequently deliver and report the study.

In this section, we outline three studies that have evaluated the uptake of a specific COS or a list of standard outcome measures, and, later, we conclude this chapter with suggestions for how this evidence base might be strengthened. The two studies that have assessed the uptake of a COS focussed on the field of rheumatology [169, 170], whilst the study of standard measures is from maternity care [171].

The first study evaluated uptake of the World Health Organisation/International League of Associations for Rheumatology core set of endpoints for use in randomised trials of treatments for rheumatoid arthritis [169]. This COS was established in 1994 following the 1992 OMERACT (Outcome Measures in Rheumatology) conference and consisted of seven measures (pain, patient global assessment, physical disability, swollen joints, tender joints, acute phase reactants and physician global assessment) and one additional measure (radiographs of joints) for studies lasting more than 1 year. The study explored trends in the choice of outcomes that were reported in rheumatoid arthritis trials over two decades and also aimed to identify reasons that the COS was not implemented in trials that did not measure the full COS after it had been published. The sample of 350 randomised trials was identified from those included in 48 Cochrane reviews of rheumatoid arthritis. The report for each of these trials was read to ascertain whether or not the items in the COS had been measured or reported. If the trial had not measured all the outcomes in the COS, the correspondence author was emailed to determine their awareness of the COS and their reasons for not adopting it in their randomised trial. Identifying whether or not the RA COS was measured in the cohort of RA trials was a straightforward process, mostly requiring only a review of the ‘outcome assessment’ paragraph of the ‘Methods’ section of a trial report, but time-consuming since reports needed to be obtained.

There was an increase in the proportion of randomised trials measuring the COS items over time, with almost 70% measuring all these outcomes in trials that were published at the end of the first decade of the twentieth century. Of the trialists who did not measure the full COS, the survey revealed that most were unaware of the COS when selecting outcomes to measure. Amongst those who were aware of the COS, two did not measure all of the outcomes because their trials focussed mainly on safety, one did measure all outcomes but failed to report one of them and one had already selected the outcomes for their trial before the COS was published.

The second study to assess COS uptake considered the ASAS (Assessment of SpondyloArthritis International Society)/OMERACT axial spondyloarthritis (AS) COS for disease-controlling anti-rheumatic therapy (DC-ART) and symptom-modifying anti-rheumatic drugs (SMARDs) [170]. Both COS include physical function, pain, spinal mobility, spinal stiffness, fatigue and patient global assessment. In addition, the DC-ART COS includes peripheral joints/entheses, acute phase reactants and radiographs of the spine. The study assessed uptake of the outcomes from these two COS in randomised trials of pharmacological and non-pharmacological interventions in AS.

A total of 99 randomised trials were identified by a systematic review and separated into those reported in the first 2 years after publication of the COS (*n* = 48) and those reported at least 2 years after the COS (*n* = 51). The study found that some of the AS COS outcomes were more frequently measured in the second group of randomised trials. These included physical function, peripheral joints/entheses and fatigue in the DC-ART COS and physical function and fatigue in the SMARD COS. However, there was a decrease in the frequency for one of the SMARD COS outcomes: patient global assessment. Overall, 20% of randomised trials published at least 2 years after the COS measured all outcomes included in the COS.

Finally, the third study looked at gestational diabetes mellitus (GDM) research to assess the uptake of ‘standard outcomes’ that had been developed by the WOMBAT (WOMen and Babies health and wellbeing: Action through Trials) Collaboration [171]. The study surveyed Cochrane protocols and reviews that examined interventions for GDM before and after the ‘standard outcomes’ were made available by WOMBAT in 2009. Four protocols and 13 reviews (including 49 trials, published from 1983 to 2012) were included. One protocol and five reviews were published during 2001–2009 and three protocols and eight reviews were published during 2010–2014. These covered prevention (5 protocols/reviews), detection (2), management (8) and follow-up (2). There was reasonable consistency between the protocols and reviews from before and after 2009, with a mean of four prespecified primary outcomes, covering 12 different maternal outcomes and nine different child outcomes. The most common primary outcomes for mothers were GDM and caesarean birth. The most common outcomes for their children were perinatal mortality, large-for-gestational age and macrosomia. The number of prespecified secondary outcomes was higher after WOMBAT’s ‘standard outcomes’ were made available (means of 26 and 48 in 2001–2009 and 2010–2014, respectively). There was an increase in the number of outcomes that were prespecified by at least half of the protocols and reviews, with this being particularly true for those related to longer-term follow-up.

### 3.3 Implementation

Although there is a paucity of research into the uptake of COS, it is clear that there is still room for improvement in their uptake and implementation. Several different actors have a role in this and are already seeking to improve matters.

#### 3.3.1 Role of the COMET Initiative

The COMET Initiative is facilitating the uptake and implementation of COS [21], and seeking to avoid the waste that might be generated by unnecessary duplication and a proliferation of COS, by:

1. Making it easier for COS developers and potential users to find COS through the free availability of summaries of ongoing and completed COS in the COMET database [22, 38]
2. Highlighting new COS in a news feed on the COMET website
3. Encouraging COS developers to produce an implementation plan from the start, including consideration of any potential barriers and cost implications
4. Working with organisations that cover multiple areas of health and social care, including academic initiatives such as Cochrane, SPIRIT (Standard Protocol Items: Recommendations for Interventional Trials), GRADE (Grading of Recommendations Assessment, Development and Evaluation) Working Group and researchers involved in the development of COS; as well as potential funders of COS development and of research more generally, industry, health care regulators, developers of guidelines and journals

The first two of these activities are covered in Chapter 2 about the COMET database, and the present chapter includes a section highlighting issues that COS developers might consider when developing an implementation plan (see below). Some examples of the work of the COMET Initiative towards the fourth are outlined here.

#### 3.3.2 Role of core outcome set developers

Little is known about how COS developers perceive their role in implementation. In a recent survey, researchers who had published COS were asked ‘Was the future implementation or uptake of the core outcome set considered by your group at any stage?’ [65]. Of 76 responders, 12 (16%) had not, indicating either that they saw implementation as beyond the scope of COS development, or that funding was not available to support the implementation work. Those developers who had considered this task listed the following as implementation activities: publication in a journal (*n* = 18), participation in meetings (*n* = 11), talking to relevant stakeholder groups (*n* = 7), the involvement of prospective users in the development process who might influence uptake later on (*n* = 5), uptake in guidelines (*n* = 3).Those developers who had considered this task seemed to focus their answers more on dissemination than implementation, reflecting the conflation of these two different topics which also appears elsewhere in health research. For instance, 18 responders listed publication in a journal (*n* = 18). Some reported a more proactive role in trying to get their COS implemented beyond their own research team; and although it remains unclear whether some of these activities should be categorised as dissemination or attempts to ensure implementation, they included participation in meetings (*n* = 11), talking to relevant stakeholder groups (*n* = 7), involving prospective users in the development process who might influence uptake later on (*n* = 5), and uptake in guidelines (*n* = 3).

##### 3.3.2.1 Stakeholders as future implementers

As well as developing an implementation plan for what they would like to happen with their COS (see below), COS developers should consider how the development process itself might help with the subsequent implementation of the COS. For example, the international nature of the stakeholders who are involved in its development and the various groups and disciplines that are brought together may have an impact on the perceived relevance of the COS to countries and groups that are represented, or are not represented.

Furthermore, the actual involvement of individuals in the development may influence their subsequent uptake of the COS, as has been shown previously with the results of clinical trials [172]. This is being examined in a randomised trial and observational cohort study embedded within the development of a COS for studies of mechanical ventilation in critical care. COVenT (Core Outcomes in Ventilation Trials) includes a prospective observational cohort study to determine if participation of trial investigators in the Delphi panel for the COS increases subsequent COS uptake, on the basis of the use of the COS in published reports of clinical trials in the decade after the COS is published. Authors of studies of mechanical ventilation were identified from extensive literature searches and were randomised to either participate in the Delphi panel as a ‘trial investigator’ stakeholder representative or not to participate. Five and 10 years after their COS is published, the COS developers will examine the publications of all these researchers to compare the use of the COS between the two groups [132].

##### 3.3.2.2 Development of an implementation plan

The implementation plan for a COS should reflect the particular circumstances of the COS and the area of health or social care for which it was developed. It might be that the COS was developed with a particular focus on helping with the design of a new randomised trial [173], but more generally, it is likely that the COS was developed with the intention of improving research more widely. With this in mind, COS developers could consider the following elements for their implementation plan:

1. Register the intention to do the COS with the COMET Initiative, include an entry for it in the publically accessible database when work begins and ensure that the final COS is linked to from the database
2. Disseminate the COS to researchers in the area of health or social care, through publication in an appropriate journal and presentation at relevant conferences
3. Contact funders of research in the area of health or social care, relevant Cochrane Review Groups, guideline producers, regulators and relevant commercial organisations to let them know about the COS
4. Inform those responsible for planned and ongoing research identified through prospective registries, including trial registries such as those accessible through the WHO portal for international clinical trial registries [174] and PROSPERO for systematic reviews [175]
5. Contact journals in the area of health or social care to suggest an editorial or commentary about the COS

#### 3.3.3 Role of trialists

As part of the planning for their trial, researchers who wish to maximise the potential impact of their findings need to ensure that they will measure outcomes that will make it easier to show the relevance of those findings. They can do this by determining whether or not a COS exists and then considering whether it might form the basis for the outcomes to measure in their trial. This would not restrict them to the COS alone, but provides them with the minimum to include. They remain able to include additional outcomes, especially those that might be particularly pertinent to their study. This is noted in the SPIRIT (Standard Protocol Items: Recommendations for Interventional Trials) guidance on the reporting of protocols for clinical trials, which recommends ‘where possible, the development and adoption of a common set of key trial outcomes within a specialty can help to deter selective reporting of outcomes and to facilitate comparisons and pooling of results across trials in a meta-analysis. The COMET (Core Outcome Measures in Effectiveness Trials) Initiative aims to facilitate the development and application of such standardised sets of core outcomes for clinical trials of specific conditions [34]. Trial investigators are encouraged to ascertain whether there is a COS relevant to their trial and, if so, to include those outcomes in their trial. Existence of a common set of outcomes does not preclude inclusion of additional relevant outcomes for a given trial’ [176].

In deciding on the relevance of a COS to their trial, researchers should consider its scope, defined in terms of the health condition, target population and whether it is for any intervention or specific types of intervention. These cover the first three elements of a PICO (Population, Intervention, Comparator, Outcomes) structured question for a clinical trial, and it may be that an existing COS helps a trialist to decide on the outcomes to measure based on a partial overlap between some of these elements in a COS and those for their trial. Once again, the availability of hundreds of COS in the COMET database makes it much easier for researchers to identify all the COS that might be useful to them, without the need to do their own extensive searches of the academic literature [177].

#### 3.3.4 Engagement with funders

The importance of COS is increasingly being recognised by research funders. For example, the National Institute for Health Research (NIHR) in the UK, the Health Research Board in Ireland and the charity Arthritis Research UK, all highlight COMET to researchers seeking funding for new trials and studies. In the UK, the NIHR Health Technology Assessment (NIHR HTA) Programme includes the following text in their *Guidance Notes for Completing Full Proposals*: ‘Details should include justification of the use of outcome measures where a legitimate choice exists between alternatives. Where established Core Outcomes exist they should be included amongst the list of outcomes unless there is good reason to do otherwise. Please see The COMET Initiative website [34] to identify whether Core Outcomes have been established’.

#### 3.3.5 Engagement with prospective research registries

The benefits of prospective registration of research are now widely recognised and accepted [174]. The registration process provides an opportunity to encourage researchers to consider the use of a COS at early stages in the planning of their study. For example, in planning their trial and preparing their entry for the trial registry, researchers should identify whether a relevant COS already exists and, if so, they should be explicit about whether or not they will use it. If they will use it, they should cite the COS and specify each of the outcomes in the COS and any additional outcomes that they intend to measure [178]. By listing the COS outcomes, the researcher will make it easier for users of the registry to see what they will measure and to find their entry if they search on outcomes. One way to make this easier to achieve would be for the trial registry to link to registries of COS that would allow the entry for a trial to be populated automatically with the COS outcomes (which might then be edited by the researcher, if necessary). If a researcher finds and considers a relevant COS when designing and registering their trial, this might also help them in choosing and justifying their primary outcomes [179]. Primary outcomes are likely to be used in calculating the sample size for the trial, and should also be those that are given the highest priority for measurement and reporting by the researcher, with particular efforts to minimise missing data. When a trial has closed, the registry entry remains important for those who wish to use the trial in decision-making because it can help them to decide if any reports of the trial are subject to selective reporting of outcomes. If the outcomes in a COS were listed in the registry entry, systematic reviewers and others who wish to use the trial’s findings should find it easier to assess the risk of selective reporting bias.

Similarly, systematic reviewers might themselves make more use of COS, and be clear about doing so. They should consider whether to use a COS when choosing the outcomes to use to test the effects of the interventions that they will investigate in their review [180]. As with trials, this use of a COS should be clear in the protocol for the review and in its entry in a prospective registry. Cochrane, as the world’s largest producer of systematic reviews has, since its inception in the 1990s, required those preparing Cochrane reviews to register their proposed review with the relevant Cochrane Review Group before preparing the protocol for their review which would be published in the Cochrane Database of Systematic Reviews following editorial approval. This process relates only to Cochrane reviews but, since 2011, all systematic reviewers in health care have been able to prospectively register their review in the free-to-use, publically available registry for systematic reviews, PROSPERO. This was developed by the Centre for Reviews and Dissemination in the University of York, UK [175] and, as of 2017, contains more than 20,000 entries for systematic reviews. The researchers are able to list the outcomes that they wish to use in the review and, as with trialists, they could include the COS, the specific outcomes within this and any additional outcomes that they are interested in. Just as a COS might help a trialist to choose their primary outcomes, reviewers might find that a COS helps them to choose the outcomes to include in a Summary of Findings (SoF) table (as noted above) [179] and to focus their efforts on obtaining data for these outcomes, even if the data are not readily available in the reports of eligible studies. Similarly, the explicit use of a COS in the entry for a review will help users to decide if the review is affected by selective reporting bias if all the outcomes from the COS are now tackled in the review’s results [181].

#### 3.3.6 Engagement with journals

COS have been published in a range of journals over the last two decades [14, 39, 177] and some journals now publish the protocols for the development of COS. This will help to raise awareness of COS and, coupled with the COMET database, will improve access to them. However, as with the uptake of guidelines for the reporting of different types of research study, such as CONSORT for randomised trials [182] and PRISMA-IPD for the reporting of meta-analyses that used individual participant data [183], the next step will be the active encouragement by journals of the use of COS in research studies. An important recent development along these lines is the CROWN (Core Outcomes in Women’s Health) Initiative. This is a consortium of over 78 obstetrics and gynaecology (and related) journal editors which will ‘strongly encourage the reporting of results for COS. Facilitate embedding of COS in research practice, working closely with researchers, reviewers, funders and guideline-makers’ [184].

#### 3.3.7 Role of systematic reviewers

Systematic reviewers are often the researchers who struggle most because of inconsistencies in the outcomes that have been measured and reported in trials of health and social care. This hampers their ability to bring together the evidence from research that already exists, and to compare, contrast and combine it to resolve the uncertainties that they are addressing in their review. Alongside key stakeholders, such as practitioners, patients and others users of health and social care services, researchers, policy-makers and those who fund services and research, there is an important role for systematic reviewers in the development of COS. Their involvement may help to overcome some of the problems encountered by reviewers when trying to select outcomes for their review and by users when reading reviews.

Currently, the explicit use of COS in systematic reviews seems rare. The aforementioned study of Bain et al. assessed the use of the WOMBAT standard measures in Cochrane protocols and reviews for gestational maternal diabetes [171] and fuller surveys of all Cochrane reviews that were first published in 2007 (387 reviews), 2011 (401) and 2013 (439) investigated the choice of outcomes more generally [101, 185]. This research found that none of these 1227 reviews cited a COS when discussing the choice of outcomes investigated in the review. The reviews included a total of nearly 9800 outcomes, with the most recent sample (reviews published for the first time in 2013) ranging from three reviews that assessed just one outcome measure to a review with 62 outcome measures. The median number of outcomes was seven. As noted above, COS might be particularly useful for Cochrane reviews during the selection of outcomes to include in the SoF developed by the GRADE Working Group to summarise the results for important outcomes and the quality of this evidence. These tables were introduced to Cochrane reviews in 2008, and were included in 112 (31%) of the 361 full reviews published for the first time in 2011 that contained at least one included study, rising to 57% (216 of 375 reviews) in 2013 [185].

#### 3.3.8 Engagement with Cochrane

Cochrane is the world’s largest organisation dedicated to the preparation and maintenance of systematic reviews in health and social care. More than 9000 protocols and full reviews have been published in the *Cochrane Library* over the last 20 years and COMET has worked with Cochrane on a variety of projects to facilitate the uptake of COS in these reviews. This has been done partly in the context of the SoF tables that are now included in many of these reviews [186]. These tables present the results of the review for up to seven outcomes that are deemed to be important to patients, to improve the understanding and retrieval of the key findings of the review.

In 2011–2012, alongside an investigation of the amount of missing patient data for primary outcomes in Cochrane reviews, the coordinating editors of Cochrane Review Groups (CRGs) were surveyed about issues related to the standardisation of outcomes in their CRGs’ reviews [16]. Almost all the coordinating editors (from 45 of the 50 CRGs surveyed) responded to this survey, revealing that 14 coordinating editors (31%) had been involved in the development of a COS and 16 (36%) were aware of other work to develop a COS for conditions relevant to their CRG. It also found that 16 (36%) CRGs had a centralised policy regarding which outcomes to include in the SoF table and 33 (73%) coordinating editors thought that a COS for effectiveness trials should be used routinely for a SoF table. The coordinating editors listed advantages and challenges associated with standardising outcomes across reviews in their CRG, with the most common advantage for having a COS being that systematic reviews or meta-analyses would benefit from the standardisation of outcomes. The main challenges were the processes needed to develop a COS, how to decide when a COS should be applied and the persuasion of potential authors of Cochrane reviews to use the COS. Since this survey, discussions with Cochrane have been ongoing about the use of COS in Cochrane reviews. In particular, as the proportion of Cochrane reviews with a SoF table is increasing, COMET is in discussion with those responsible for the guidance on these (including GRADE) about emphasising the potential value of COS when selecting the up to seven outcomes to include in one of these tables.

### 3.4 Review and feedback

Assessments of the uptake of COS, such as the few that have been done to date (see above) and those that could be done as part of a wider research agenda (see below) can provide an opportunity to review an existing COS. If these investigations reveal that randomised trials or systematic reviews are not measuring or assessing a particular outcome that is included in the COS, the relevance of that outcome to the COS might be reconsidered. Likewise, given the recognition that trials and reviews are likely to continue to study outcomes outside a COS, if the research studies in a particular area of health or social care are consistently measuring an outcome that is not included in the COS, a revision or update of the COS might be indicated.

Decisions about whether a COS is ‘fit for purpose’ might be informed by interviews with the intended users of the COS, such as trialists, to gather their reasons for implementing a COS or for not doing so. This can provide an understanding of the decision-making process that they used when choosing which outcomes to measure and the value that they place on COS developed by others. This may suggest improvements to methods of COS development; for example, including a particular stakeholder group that would encourage more trialists to adopt a COS in their trials.

COS developers should consider opportunities to review their COS periodically. Some groups, such as OMERACT, already have mechanisms in place to review their COS which have led to important changes. For example, the development of the original OMERACT COS in rheumatoid arthritis was done without direct patient input; but focus groups with patients were held at the 6th OMERACT meeting in 2002 to build upon the findings from a previous email survey. The survey had identified fatigue and sleep as missing from the OMERACT COS and an analysis of the patient focus group discussions strengthened further the importance of fatigue which was then added to the COS.

Given the low proportion of existing COS that have involved patients to date, it may be expected that a future review of such a COS would identify patient input to be an important gap. Where an existing COS has been developed without the perspective of patients or other key stakeholders, qualitative studies may help to address this omission.

Several examples exist where periodic reviews were planned in advance. In 1991, guidelines for controlled trials of drugs in migraine were published [187]. These guidelines included recommendations about the outcomes that should be measured, and were based on the experience of the committee members and in some cases on analysis of previous trials. They stated that a revised second edition of these guidelines was planned for 3–4 years’ time. A second edition of these guidelines was subsequently published in 2000 [188]. They stated that ‘The experience of clinical investigators and the pharmaceutical industry has expanded enormously, providing a basis for revising these guidelines now’. A third edition of these guidelines was published as a ‘consensus summary that was developed by experts in the field’.

The International Headache Society Committee on Clinical Trials also published guidelines for trials of drug treatments in tension-type headache [189]. They similarly stated that a revised second edition of these guidelines was planned for 3-4 years’ time, subsequently published in 2010 [190]. They stated that ‘Since 1995 several studies on the treatment of episodic and chronic tension-type headaches have been published, providing new information on trial methodology for this disorder’.

In another example, a consensus conference in ovarian cancer involving 52 experts produced recommendations about study methodology including outcomes for trials [191]. It was recommended that they reconvene in 4 to 5 years to review the latest evidence from high-quality clinical trials [192]. There was a proceeding consensus conference, following the same methodology, which was attended by 77 experts. They stated that ‘This conference served to further advance the knowledge provided in earlier such conferences and to provide new parameters for trial design,’ which specifically included the consideration of patient-reported outcomes.

Additional work has been undertaken following the publication of a COS for vitiligo. The original objective was to create guidelines for randomised controlled trials investigating interventions used in the management of vitiligo, and the work was based on the assessment of the methodology of RCTs included in a 2010 update of the Cochrane systematic review ‘Interventions for Vitiligo’ [193]. The guideline developers included authors of the Cochrane review (clinicians, patient representatives and a statistician) plus the coordinator of the vitiligo priority-setting partnership at the Centre of Evidence-Based Dermatology at the University of Nottingham. Subsequent work was carried out specifically around outcomes, including a systematic review of outcomes and a survey of patients and clinicians on outcomes in vitiligo trials [194]. This, in combination with the original publication, was described as ‘a good starting point for creating a consensus on a core outcome set for vitiligo trials’. A Delphi study was carried out with the aim of developing ‘international consensus over a COS for vitiligo trials that is acceptable to health care professionals, patients and their caregivers, researchers and regulatory bodies’ [195]. Further work is now underway to develop definitions of outcomes, and determine the best way of measuring recommended outcomes.

Periodic review would allow developers to confirm the ongoing validity of the COS, ensure that included outcomes are still relevant and important, and that no excluded outcomes should be added. It would also allow them to evaluate how successful implementation has been and to engage further stakeholders as appropriate. However, as with the updating of systematic reviews [196], there are uncertainties about the frequency with which this re-visiting should be done, and whether there might be triggers to prompt it.

### 3.5 Future research into the uptake and implementation of core outcome sets

We conclude this chapter with some suggestions for research that could help to determine the uptake, implementation and impact of COS. This draws on the approaches taken in the small number of existing examples of such research, as noted above, and on ideas around assessments of knowledge transfer and research impact more generally.

#### 3.5.1 Clinical trials registries

As noted above, researchers are encouraged to consider COS when registering their clinical trials or systematic reviews [178, 180]. Registries provide public access to information on planned, ongoing and recently completed trials and reviews. An assessment of the listed outcomes would provide an up-to-date guide to the outcomes that the researchers intended to measure or assess. It would also provide a way to determine the proportion of current trials and reviews in a particular area of health that are using the appropriate COS.

#### 3.5.2 Applications for funding and ethical approval

In some cases, an even earlier step than trial registration may be the researcher’s application for funding or ethical approval to do their study. Access to such applications would allow an assessment of the impact of encouragement to use COS by funders such as the NIHR HTA Programme (see above), as well as the uptake of COS regardless of such encouragement. Such assessments could use methods that have been employed previously to look at cohorts of funding applications and submissions to ethics committees to examine, for instance, the use of systematic reviews in the design of the proposed studies [197–199].

#### 3.5.3 Citation analysis and surveys of reports of trials and reviews

Moving on to the published reports of research, at its simplest, citation analysis involves counting the number of citations received by a COS publication, author or research team, taking account of the volume of their research output [200, 201]. The assumption is that the higher the citation count, the greater the impact of the research, and this type of study can be relatively quick given the existence of citation databases. However, simply counting the number of citations loses the subtlety of the context for the citing of an article and, so, a more in-depth citation analysis would look at the citing documents to try to determine the reason for the citation [200]. Further refinements of this type of research might include a focus on the citations of the COS article in reports of randomised trials or systematic reviews if these were the principal targets that the COS was developed for. Such work might also include qualitative research to explore why trialists and reviewers did, or did not, use the COS; akin to that done in the aforementioned study of the uptake of the OMERACT COS [169].

More generally, assessments could be done of collections of reports of trials and reviews to see whether these describe the use of COS and to survey the outcomes that have been used. The latter is often a first step in the development of a COS, providing the basis for the list of outcomes that might be considered in the early stages of a consensus process such as a Delphi exercise [14]. As noted above, such assessments of impact have been done for reports of randomised trials in relation to the OMERACT COS [169], Cochrane protocols and reviews of gestational maternal diabetes [171] and new Cochrane reviews from 2007, 2011 and 2013 [101, 185].

### 3.6 Conclusions

The development of a COS should not be seen as an end in itself, but rather as a means for making it easier for researchers in a particular area of health or social care to do better and more informative studies that will resolve important uncertainties. Therefore, the various actors in the development of COS and potential users have roles to play in ensuring that the COS are considered when new research is being planned. This should lead to greater uptake and implementation, and provide opportunities to keep the relevance of existing COS under review. The development of COS should also be subject to rigorous evaluation to examine the impact of COS, and to identify the most effective and efficient methods for their future development and enhancement.

## Chapter 4: Discussion

This Handbook describes what we currently know about the methods for COS development, implementation, review and uptake. There is no ‘gold standard’ method for COS development. For some aspects, we have recommendations for practice; for others, we have identified issues to consider; and in other areas we have highlighted the need for research. In this chapter, we discuss these proposals for the future of COS development and uptake.

### 4.1 Recommendations for practice

Researchers should clearly define the scope of the COS to be developed.

COS developers should register their project in the COMET database.

A protocol should be developed to describe the work that will be done to develop the COS, and made publically available.

A COS implementation plan should be considered from the outset, including how the development process might help to improve subsequent uptake.

COS developers should identify and take into account relevant existing knowledge as part of the development process. Sufficient time should be given to structuring and wording any initial list of outcomes to be considered in the process.

If a Delphi study is undertaken as part of the consensus process, feedback should allow all stakeholder groups to see the results from other stakeholder groups separately before re-scoring. This follows evaluation in several nested randomised studies which show that providing feedback separately by stakeholder group improves the degree of consensus reached [130].

The final COS should be agreed following one or more face-to-face meetings involving relevant stakeholders groups, organised in order to discuss the results of prior work, including the review of existing knowledge and the establishment of stakeholder opinions.

The COS development study should be published following the COS-STAR reporting guideline.

Researchers should use the COSMIN-COMET guidance to determine how to select instruments for measurement of outcomes in a COS.

### 4.2 Recommendations for research

The credibility of a COS depends on the use of sound methodology in its development, and, transparent reporting of the processes adopted. The implementation of the COS in clinical trials will depend on effective dissemination and its acceptance by the research community which includes researchers, patients and the public and funders. To improve the methodology, reporting and implementation of COS, further research is required to address the following objectives:

1. *To compare different methods used to develop COS, with respect to minimising bias, maximising efficiency, and increasing the degree of uptake*
  Interviews with COS developers revealed that they would have appreciated methodological guidance early on in the COS development process (Gargon et al. 2016). Our systematic review revealed wide variation in the methods used to develop COS. We have identified a wide range of research that is needed to provide a robust evidence base for the methods associated with COS development. Work is needed to compare existing methods in order to identify ways to minimise bias, maximise efficiency and increase uptake, thereby reducing waste. In order to improve the methods, we recommend that COS developers take the opportunity of nesting methodology research studies, whenever possible, in their work to help resolve uncertainties and strengthen the evidence base. This evidence base can then be used as a basis for recommendations for practice, as is the case with the evaluations of the methods of feedback to different stakeholder groups, which led to the recommendation for practice above.
2. *To understand better the patient perspective and strengthen patient and public engagement in studies developing COS*
  COS need to include those outcomes that are most relevant to patients and carers, so it is vital that patients and carers are involved in their development. There are examples of where involving patients in the process identified an outcome that was important to them as a group and was subsequently included in the COS, but which might have been overlooked if the COS had been developed by practitioners on their own.
  Researchers are increasingly including patients and the public alongside other stakeholders in identifying what outcomes to measure in clinical trials, but uncertainty remains about the best ways to do this. For example, although only 22% of COS published up to 2014 reported that there was input from patients in their development (Gargon 2015, Gorst 2016), nearly 90% of ongoing studies include patients as participants. The uncertainty is no longer around whether patients should participate, but rather the nature of that participation.
3. *To develop a consensus-based guideline for the content of a COS development protocol*
  It is recommended that a protocol be written for COS development studies. A guideline for the content of this protocol, similar to the SPIRIT guidance for the content of a clinical trial protocol [176], and developed through a consensus process, would be helpful.
4. *To develop and disseminate advice regarding issues to consider when assessing studies developing COS*
  To date, there has been no formal quality assessment of COS studies, and the COMET database includes all those that have been identified as meeting the eligibility criteria but without any central assessment of quality. However, defining the quality of a COS is not straightforward. In principle, a ‘good’ COS is one that is implemented and leads to improved outcomes for patients but this might be far down-stream of the development process. Work is needed to assess the implications of the different methods of COS development for both minimising bias, maximising efficiency, and increasing uptake on the likelihood that the COS will have this positive impact.
  Even if robust methods are identified for developing COS, whether these methods were followed might not be easily assessed from a report of that process. However, the report might allow the potential user of the COS to consider how the developers minimised biases that can occur in the process, particularly if the COS-STAR reporting guideline is followed. There is a pressing need to assess how a COS has been developed using internationally recognised minimum standards that are valid and reliable and need to be agreed. This is particularly important for the research community who are considering whether or not to use a COS, in order to allow them to decide whether an existing COS is good enough to be adopted. In some cases, they might need to decide between different COS that may be relevant to their specific research study. This research to identify the key elements in the COS development and reporting process would feed into the future review of the COS-STAR reporting guideline.
5. *To compare different methods for disseminating, improving access to, and promoting the use of, COS on their uptake in trials and reviews*
  Previous research has identified perceived challenges to the widespread implementation of COS [16]. These include slow or limited uptake, multiple groups developing COS for the same topic and inconsistencies between these COS, accessibility of relevant COS to all stakeholders, and the need to update COS over time. Several methods have been suggested to improve the uptake of COS, including improved dissemination and accessibility (such as through the COMET database and better indexing of reports of COS), pressure from funders in the drive to reduce waste in research [202], and advocacy by patients and the public. Future developers and funders of COS would benefit from evidence on the effectiveness of different strategies and how best to implement these.
6. *To identify best methods for disseminating COS to patient participants and the wider patient community*
  It is good practice to share the results from the COS study with everyone involved in decisions about health and social care, including patients. There are few examples of this, however, which is likely to reflect the limited involvement of patients to date in this area of research. Examples of involving public research partners in writing the end-of-study information, are beginning to emerge [79] but more work is needed to understand optimal forms of communication and dissemination in particular settings.
7. *To examine the level of uptake of existing COS and understand the reasons why researchers (including trialists and systematic reviewers) do, and do not, use them*
  Although a small number of studies have directly assessed the impact of individual COS on clinical trial design [169, 170], more research is needed to investigate whether, or how, COS might make a difference to trials and reviews. It is timely to examine the level of uptake of a wider range of existing COS and to understand the reasons why researchers working on trials and reviews do (and do not) include the recommended outcomes in their studies.
  For example, our survey of more than 1000 new Cochrane reviews from 2007, 2011 and 2013 found that none of these made explicit reference to a COS in their design or results. Research is needed to understand the attitude of researchers to the use of COS in systematic reviews, to identify barriers and facilitators, and to repeat and extend the survey to assess uptake in this community.
  Research into the uptake of COS should make use of existing knowledge from the field of implementation science to understand how to effect professional behaviour change in this context. Awareness of COS and COMET is growing through advocacy, with a recommendation to use a relevant COS being made in guidance for trialists [176], applicants for grant funding (for example, NIHR, see ‘Implementation’ in Chapter 3 above’), applicants for regulatory approval [203], clinical guideline developers (Chapter 4.3 in [204]).
  Reviewing lessons learnt from experience of other initiatives providing guidance to trialists will be useful. For example, CONSORT is very widely known, and whilst some aspects, such as the inclusion of the flow diagram, have had a major impact [205], overall adherence remains disappointingly poor [206]. This shows that whilst it is possible to achieve change, it is difficult and certainly slow. The analogy with CONSORT is not exact however. COMET addresses study design and so there is potential for influence at the initiation of research including the funding stage. CONSORT, by contrast, whilst relevant to consider at the design stage is primarily relevant after the research is completed and is being written up. This may be an important difference.
8. *To understand better the role and contribution of qualitative research in COS development*
  The use of qualitative research in COS development is an evolving area and there is only limited experience of qualitative research in this context. There are several areas where methodological work around qualitative research in the context of COS development would be helpful, including:
  Use of existing qualitative research and secondary analysis of qualitative data as part of the foundations for the COS development
  Use of qualitative research at different stages of COS development, including to update existing COS
  Methods of qualitative data collection and of eliciting stakeholder perspectives on outcomes
  Impact of qualitative research on stakeholder participation in subsequent processes
  Impact of qualitative research on user confidence in COS

### 4.3 Other applications for core outcome sets

#### 4.3.1 Core Information Sets

Patients require information in order to participate in decision-making and provide their consent to treatment. The amount of information that could be communicated is large and it is often unclear what information is most important for achieving understanding of treatments and their consequences. There is a danger of overwhelming patients with data and technical detail which may hamper their understanding and increase anxiety. Furthermore, clinicians may vary the information provided, thus reducing consistency of practice. The importance of this issue has been highlighted in a recent landmark ruling by the UK Supreme Court. Now, the law is aligned more closely with professional standards that require clinicians to spend time with patients discussing the risks, intended benefits, and reasonable alternative options before seeking consent to proceed. Within a process of shared decision-making it is expected that the physician and patient agree together on an appropriate treatment plan that fits the patient’s values and situation. The physician is responsible for finding out what risks associated with the treatment matter to a reasonable person in the patient’s position or would probably matter to the individual patient [207]. One new and developing method for physician to use in clinical consultations is to identify ‘core information’. This includes information of importance (that is valued) by patients within a ‘core information (disclosure) set’ [208]. Core information represents the minimum information to be discussed in all consultations for a particular intervention. The idea of core information was described over 30 years ago when ‘core disclosure’ was recommended [209]. It was suggested that a core disclosure set would include information of importance to key stakeholders (patients and clinicians), be feasible to communicate in a regular clinical consultation, and act as a stimulus for further discussion of importance to the patient. Although a seminal idea, it has received little attention or application until recently when clinicians involved in the COMET Initiative have adapted core outcomes methodology to develop Core Information Sets [210]. Core Information Sets for surgery for oesophageal [58] and colorectal cancer have been developed and more recently for head and neck cancer. More work is still needed to establish how Core Information Sets are optimally used in clinical consultations and which outcomes best assess their effectiveness at improving informed consent.

#### 4.3.2 Core outcome sets in routine care

The International Consortium for Health Outcomes Measurement (ICHOM) organises global teams of physician leaders, outcomes researchers and patient advocates to define core sets of outcomes per medical condition for use in clinical practice rather than clinical trials. Health care is complex and medical knowledge is changing fast. Reliable outcome data enable physicians and patients to make better decisions about what treatments are best for them and who should provide them. ICHOM is a relatively new initiative, aiming to publish 50 standard sets by 2017. A list of completed sets, in progress and conditions under consideration can be found on the website [211]. ICHOM has registered completed projects in the COMET database after the COS has been agreed. There has been a call for more transparency in the reporting of methods used by ICHOM to develop these core sets for routine care [212].

The continuous measurement of health care quality is an essential aspect for the establishment of a high quality of care [213, 214]. Quality indicators are used to measure structures, processes, indication and outcomes of patient treatment [215]. National audits often include assessment of data on patient outcomes, and COS may be of relevance here also.

Due to the large number of quality indicators and their heterogeneity, a global comparison even within medical disciplines is hardly possible. For example, an audit of the clinical guidelines from the National Institute for Health and Care Excellence (NICE) found that a total of 1795 quality indicators were used [216]; in the German health care system, there are nearly 2000 [217] and in other health care systems, such as the USA or Canada, up to 700 different quality indicators [218, 219] have been recommended.

Core outcome sets could be a powerful, science-grounded strategy to derive patient-relevant outcome quality indicators and to obtain meaningful results to compare quality of care between providers, regions, nations and health care systems and thus to stimulate value-based health care and the development of health care systems. However, the methodological quality for the development of most quality indicators is unclear [220, 221].

Although major features of the established methods to develop COS for trials also apply to other settings, the requirements for measurement instruments to be recommended by a core set may differ between trials and routine care or quality measurement.

#### 4.3.3 Patient registries

Patient registries have been established for a variety of reasons, including disease surveillance, studying the natural course of a disease, post-market surveillance of health care interventions, evaluation of interventions and assessment of the quality of care [222]. Establishing or bringing together existing national research registries that follow the same methodology can provide a powerful research platform, allowing the collection of higher-quality prospective data.

Registries collecting the same minimum data set will facilitate comparisons and pooling [223]. Although some methodological guidelines and recommendations for the development of comparable and interoperable patient registries in cross-border settings exist [224], limited attention has been paid to the development of consensus around the COS to be measured and collected on patients within the registry. Projects are starting to appear which incorporate an objective to develop a COS for patient disease registries, following similar methodology to that described in this Handbook [162].

### 4.4 Conclusions

Interest in COS development continues to increase. To reduce waste in research, and to avoid contributing further to it, COS developers should pay attention to implementation strategies in order to increase uptake. Substantial progress has been made in several clinical areas over recent years but there are many important health conditions for which no COS currently exists or is in development.

In this Handbook, we have presented an overview of the research literature around COS development and uptake. As evidence emerges, we will amend and disseminate this guidance on COS development, the assessment of uptake, and the review of COS through periodic updates of this Handbook; whilst the latest additions to this body of evidence can be found on the COMET website.

## Additional file

Additional file 1: Open Peer Review. (PDF 260 kb)
