# Supplementary material for: The COMET Handbook: version 1.0
Source: Trials. 2017 Jun 20;18(Suppl 3):280. doi: 10.1186/s13063-017-1978-4 (PMC5499094; doi:10.1186/s13063-017-1978-4)
Supplement: Additional file 1: — Open Peer Review. (PDF 260 kb) [file 13063_2017_1978_MOESM1_ESM.pdf]

Title: The COMET Handbook: version 1.0

Reviewer 1: Christopher Morris 6 April 2017

General comments

The COMET handbook will be a useful reference for people interested in or developing core outcome sets. I was asked to review Chapter 2 but also formally reviewed Chapter 1, and subsequently chapter 4, and have only minor suggestions.

Page 7 – the first sentence in 1.1 states efficacy but p8 says focus of handbook is effectiveness trials.

Page 19 - 1.3.4 Other relevant initiatives – could also include reference to ICF core sets promoted by WHO. This section would be stronger with statements about how COMET differs or complements these initiaitves.

Page 20 – COSMIN is led by a co-author so slightly disingenuous to refer to this initiative as 'They have also developed a checklist about which measurement properties are important and standards…' COSMIN checklist focuses on PROMs, however COS will include any/all eligible means of assessing outcomes. Could it be more explicit whether COSMIN scope is extended?

Page 25 – 'There is limited empirical evidence, however, regarding whether different methods lead to similar or different conclusions.' My guess is that there will be some variation in COSs produced for specific areas due to sampling effects in studies even if exactly the same methods are used.

Page 51 – 'Adverse events (or side effects) **-** Be clear that this could include things like death, pain, etc. when they are harms rather than benefits?' Seems an odd sentence, would death, pain etc really be benefits?

There is mention of ethics approval on page 89, however I missed this initially and perhaps a separate section on ethical issues and examples/advice on seeking relevant approvals would be useful.

The qualitative section is valuable, however I wondered why it was placed so late in the chapter as it pertains more to work that would be conducted before the Delphi survey to identify candidate outcomes phrased in the ways patients/carers articulate them?

Personally I would prefer to see section 2.13.3 on Patient and public involvement early in the chapter alongside section 2.6.1.

The information provided on selecting methods for 'how to measure' is relatively slim compared that provided on deciding 'what outcomes to measure', and there are few examples provided. This may be because this is part of the process is still developing however more examples of the types of decisions being made might be useful.

Chapter 4 reads well generally and succinctly discusses recommendations, just a couple of points:

P135 – first sentence - I find this hard to comprehend, I think the sentence too long or latter part could be clearer "If a Delphi study is undertaken as part of the consensus process, feedback should either allow all stakeholder groups to see the results from other stakeholder groups separately before re-scoring or this method of feedback should be compared with other methods."

Section 4, starting bottom of p138 – it's not clear (to me) how this proposed recommendation is substantively different to the Cos-Star reporting guideline. I suspect it is but just not clear how from the text.

Thanks for asking me to review this, as currently I'm writing a protocol to develop a COS and the handbook will be hugely helpful!

Reviewer 2: Elaine McColl 28/02/17

General comment

This handbook will be a useful asset for applied health researchers, triallists and users of research. I was asked to focus on Chapter 3

I would have liked to have seen some more acknowledgement, reference to and use of implementation science in this chapter. Essentially, the aim here is professional behaviour change. What theoretical underpinnings of such can be called upon? What lessons can be learnt, for example, from how reporting guidelines such as CONSORT have been implemented.

Implementation and improvement science frequently distinguishes between dissemination (publication and communication) and implementation; the former is a necessary nut not a sufficient condition for the latter. I felt that this distinction could and should have been made here.

Major compulsory revisions

I recommend re-organisation of sections 3.3.2 to 3.3.8 into a more logical order, reflecting the order in which these entities would be engaged with in a typical study – funders first, then registries and so on.

I wondered whether having a clear (and memorable) acronym (e.g. OMERACT) or title for a COS might facilitate its use and recognition, and make it easier to search for. There may also be implications for key-wording. Brief discussion of this ideas are recommended. Commentary in section 3.2 on how easy it was to identify whether the reviewed studies had indeed employed the COS in question would also be useful here.

There should be recognition of the likely lag time (given the 'gestation period' for trials etc) from publication of a COS to its expected uptake and appearance in registries, trial reports and systematic reviews.

Good points made in respect of linking COS to SoF in Cochrane Reviews. Currently SoF are confined to a maximum of 7 outcomes. Some commentary on the implications of a COS for a particular condition/type of intervention having more than 7 domains is needed here.

Do the authors feel that Cochrane should insist on use of a COS if one exists, or at least justification of why it is not used?

In section 3.3.4, I feel that greater emphasis needs to be placed on there being an onus on developers to publish their COS (in much the same way as it is now expected that trial protocols be published) – as recognised above, dissemination is a necessary pre-requisite for implementation. Is there a case to be made for a specialist journal?

In section 3.3.6, once again a clearer distinction could be made between dissemination and implementation.

I felt that greater and earlier prominence needs to be given to the role of PPI in COS development and how this may be achieved. Funders increasingly require the selection of patient-centred outcomes, and a COS is surely more likely to be patient-centred if patients, carers are involved in its development.

It is not entirely clear to me what point is being made in the last paragraph on page 128 and throughout page 129. Is the argument about the need for periodic revision of COS or about the importance of PPI input to COS development.

In section 3.5.2, consider suggesting that forms and checklists for peer reviewers explicitly remind them to consider whether the applicants have used a COS or justified why not.

Minor essential revisions

The first clause in the opening sentence seems odd – it is inconceivable that anyone would see the development of a COS as an end in itself, and indeed this is the conclusion drawn in section 3.6. Reworking of the opening sentence is required.

Funding bodies and journal editors need to be acknowledged as being amongst the actors at the foot of page 112.

The sentence on page 128 "Given the low … gap" is not clear and needs reworking.

Reviewer 3: John Norrie 30th March 2017

General comment

I reviewed Chapter 1 – Introduction, and Chapter 4 – Discussion. Both chapters were well written and I have only minor comments / opinions – all coming under Discretionary Revisions:

Discretionary revisions

Chapter 1 - Introduction

Section 1.1 Line 1 – 'Human beings' – do the ideas of core outcome sets extend to pre-clinical studies involving animals? And to e.g. agricultural and industrial experiments?

Section 1.1 bullet point 2. 'randomisation of persons to treatments' – is this general enough to include cluster designs?

Section 1.1 Page 8 second para – I was a bit uncomfortable with the dogma about 'The primary outcome is the outcome considered to be of greatest importance to relevant stakeholders …' – for me, effectiveness studies struggle with this straight-jacket imposed by regulatory drug trials of a single clinical primary outcome. An effectiveness study has (at least) four dimensions – does it work, is it safe, is it acceptable, and at an affordable price. So the 'primary' outcome may well be different for different stakeholders, and it should be a considered assessment of all four that decides what treatments are carried forward.

Interestingly, you then seem to back away from the certainty of a single primary outcome by then saying (Page 9) that 'Harmful effects should always be viewed as important regardless of their primary or secondary outcome label' and then in the next paragraph acknowledge the importance of cost effectiveness '… for example the financial cost associated with that outcome' – and throughout Chapters 1 and 4 you emphasise the importance of patient involvement (hence the importance of acceptability of an outcome). So in an otherwise exemplary couple of chapters it struck a somewhat bum note that in an initiative designed to promote the best outcomes (to be harsh) you seemed to promote the uncritical adoption of a single primary outcome ('the one usually used in the sample size calculation')?

Page 9. I wouldn't pick PSA as a good example of a surrogate end point! It has terrible properties, surely (at least as a screening measurement)?

1.2 Problems with outcomes. Foot of page 10 you finish describing patient-reported outcomes, top of page 11 immediately afterwards you describe line 3 'patient outcomes', which might be confusing. Suggest you just drop the 'patient' and say 'outcomes' in this latter sentence?

Page 12 'meaning that on average a new instrument had been introduced for every fifth trial' – that looked a bit odd – it assumes that the introduction of new instruments is linear, which instinctively seems unlikely. I don't think you need this statistic, personally – point is well made.

Page 13 Although I appreciate the 'reducing research waste' is an important initiative (e.g. Lancet series of publications) it is a bit negative in this context – the positive point would be that selecting the right outcomes would mean higher quality trials and getting reliable answers to improve patient health (either through primary research or meta-analyses) quicker? So it is more than 'reducing waste in research', surely?

Page 18 Populating the COMET database – I didn't understand the purpose of the first paragraph 'Sinha and colleagues …' on paediatric trials? It seemed disconnected from the more general point made in the second paragraph about the systematic review of COS. Could you either integrate it / link it in or possible delete it?

Page 19 – 'correct as of Feb 2016' – that is over a year ago?

Page 20 – perhaps be more insightful about how COMET and PROMIS will endeavour to be compatible?

Chapter 4 – Discussion

4.1. Recommendations for practice. Only COMET appears e.g. 'COS developers should register their project in the COMET database' without mentioning all the other initiatives – some of which seem relevant – discussed in Chapter 1. Should they be included or at least listed? The exception is COS-STAR but I wasn't sure what that was – was this introduced in Chapter 2 or 3?

Page 135. I didn't understand the paragraph on 'If a Delphi study …' about feedback – it wasn't clear what the alternative was, for example. Suggest you rewrite this to make it clear what the point is?

Page 136 1). I struggled a bit here on 'developers revealed that they would appreciated methodological guidance early on' and 'work is needed to compare existing methods in order to identify ways to minimise bias, maximise efficiency and increase uptake' – at first read it suggests in an unquantified way that the currents sets of COS that have been developed may in some important respects be unfit for purpose? I don't think that this is what is intended?

Page 138 This uncertainty is again underlined when the authors state 'To date, there has been no formal quality assessment of COS studies' – I think you need to either put in some quantified statement ameliorating this concern, or put it out front and say something like 'and of course such concerns on optimal methodology and quality of existing development work of COS may result in refinement or replacement of COS in future'.

And interestingly on that point, I didn't see in an obvious way any discussion of how COS will be updated as e.g. measurement standards (e.g. the development of cheap and rapid imaging modalities in a clinical area) might influence the need to change a COS?

Also – and I might have missed it – is there any specific consideration of COS in screening and diagnostic studies, rather than clinical effectiveness studies – or is that out of remit?

Page 142 – I thought the material on Core Information Sets was fascinating but isn't this a massive issue in its own right? There is a huge amount of work going on around Informed Consent and Patient Information Leaflets just in the context of randomised trials – is it sensible to get into this here?

Also, 'The importance of this issue has been highlighted in a recent landmark ruling by the Supreme Court' – I think you either have to give brief details (including which Supreme Court you are meaning) or perhaps just drop this?

Page 144 – it would have been useful to explain more fully (or exemplify) why a COS for routine care/quality measurement might differ from that for RCT. Naively, it is not obvious?

Reviewer 4: Viktoria Eleftheriadou 25/02/2017

General comments

Dear authors

Congratulations for writing such an important paper.

I have reviewed chapter 3, on implementation, review and feedback of COS. This chapter describes existing research on the uptake of COS, the most appropriate methods for the implementation of COS, followed by a discussion of the role of various factors in the implementation of COS and finally, suggestions for future research into the uptake, implementation and maintenance of COS.

I found this chapter very well structured, well written and upto the point. A hand book like this was long overdue.

Minor essential revisions

I have found a couple of typos in chapter 3. In particular:

Page 112: factors not actors

Page 116: 3.3 Implementation: Several factors (not actors)

Page 133: 3.6 Conclusions: Therefore, various factors (not actors)

Authors' response

Reviewer 1: Chris Morris

Page 7 – the first sentence in 1.1 states efficacy but p8 says focus of handbook is effectiveness trials.

> Text amended

Page 19 - 1.3.4 Other relevant initiatives – could also include reference to ICF core sets promoted by WHO. This section would be stronger with statements about how COMET differs or complements these initiaitves.

> ICF core sets are described on page 42 under 'Outcome-related frameworks'. We feel we have described COMET and other relevant initiatives clearly, and further text describing the differences would be redundant.

Page 20 – COSMIN is led by a co-author so slightly disingenuous to refer to this initiative as 'They have also developed a checklist about which measurement properties are important and standards…' COSMIN checklist focuses on PROMs, however COS will include any/all eligible means of assessing outcomes. Could it be more explicit whether COSMIN scope is extended?

> Text amended slightly. COSMIN have recently started a new project which aims to develop separate versions of the COSMIN checklist for non-PROMs. This has been added to the text.

Page 24 – 'There is limited empirical evidence, however, regarding whether different methods lead to similar or different conclusions.' My guess is that there will be some variation in COSs produced for specific areas due to sampling effects in studies even if exactly the same methods are used.

> Yes, possibly, although one might hypothesise that in studies aiming to achieve consensus that sampling effects may be less than in classical surveys.

Page 49 – 'Adverse events (or side effects) - Be clear that this could include things like death, pain, etc. when they are harms rather than benefits?' Seems an odd sentence, would death, pain etc really be benefits?

> Text has been clarified.

There is mention of ethics approval on page 89, however I missed this initially and perhaps a separate section on ethical issues and examples/advice on seeking relevant approvals would be useful.

> A sentence has been added referring the reader to guidance provided by the COMET PoPPIE group. We are trying to gather international experience of ethical approval requirements for COS work, but this is not yet sufficiently mature to be included as a section in the current version of the Handbook.

The qualitative section is valuable, however I wondered why it was placed so late in the chapter as it pertains more to work that would be conducted before the Delphi survey to identify candidate outcomes phrased in the ways patients/carers articulate them?

> Although we can see that the reviewer is referring to qualitative work likely to be undertaken before the Delphi, we thought readers new to COS (and qualitative research) might find it harder to understand the role of the qualitative work without first having sight of the sections on Delphi surveys. Essentially, we would be explaining what qualitative research can do, but it would be in the abstract. So on balance, we prefer to keep the text as it is, but we have added that qualitative work is likely to precede the Delphi.

Personally I would prefer to see section 2.13.3 on Patient and public involvement early in the chapter alongside section 2.6.1.

> We agree, it does seem better to have as much of the material as possible on patient involvement all in one place and putting it early acknowledges its importance at the start of a COS study. Text has been amended.

The information provided on selecting methods for 'how to measure' is relatively slim compared that provided on deciding 'what outcomes to measure', and there are few examples provided. This may be because this is part of the process is still developing however more examples of the types of decisions being made might be useful.

> Much of the recent work on methods for COS development that we bring together in this Handbook has focused on the 'what' to measure. As mentioned in section 2.11, a review of methods used in COS studies to determine 'how' to measure the chosen outcomes is currently underway. This can inform the next version of the Handbook in due course. The guidance developed through the COSMIN-COMET collaboration has been published in detail in Trials since we submitted the Handbook (full reference now included), so we did not feel we needed to repeat that but instead chose to summarise the principles of the approach.

Chapter 4 *reads well generally and succinctly discusses recommendations, just a couple of points:*

P135 – first sentence - I find this hard to comprehend, I think the sentence too long or latter part could be clearer "If a Delphi study is undertaken as part of the consensus process, feedback should either allow all stakeholder groups to see the results from other stakeholder groups separately before re-scoring or this method of feedback should be compared with other methods."

> Text has been amended.

Section 4, starting bottom of p138 – it's not clear (to me) how this proposed recommendation is substantively different to the Cos-Star reporting guideline. I suspect it is but just not clear how from the text.

> Text has been amended to clarify that this relates to minimum standards for COS development rather than reporting.

Reviewer 2: Elaine McColl

General comment

I would have liked to have seen some more acknowledgement, reference to and use of implementation science in this chapter. Essentially, the aim here is professional behaviour change. What theoretical underpinnings of such can be called upon? What lessons can be learnt, for example, from how reporting guidelines such as CONSORT have been implemented.

> Although the first core outcome set was published in 1981, interest and activity in the area has increased dramatically over recent years. To our knowledge, there has been no research undertaken to date on the use of implementation science to improve uptake of COS. We have added text reflecting the need to consider lessons learnt about implementation science applied elsewhere to the final chapter.

Implementation and improvement science frequently distinguishes between dissemination (publication and communication) and implementation; the former is a necessary nut not a sufficient condition for the latter. I felt that this distinction could and should have been made here.

> The first paragraph of section 3.3.6 has been revised to reflect this point.

Major compulsory revisions

I recommend re-organisation of sections 3.3.2 to 3.3.8 into a more logical order, reflecting the order in which these entities would be engaged with in a typical study – funders first, then registries and so on.

> Good suggestion, this has been done.

I wondered whether having a clear (and memorable) acronym (e.g. OMERACT) or title for a COS might facilitate its use and recognition, and make it easier to search for. There may also be implications for key-wording. Brief discussion of this ideas are recommended.

> The data available on COS uptake is currently very limited, as discussed in section 3.2. Once more data becomes available, it will be interesting to examine this hypothesis.

Commentary in section 3.2 on how easy it was to identify whether the reviewed studies had indeed employed the COS in question would also be useful here.

> Text has been added.

There should be recognition of the likely lag time (given the 'gestation period' for trials etc) from publication of a COS to its expected uptake and appearance in registries, trial reports and systematic reviews.

> Text has been added to the end of the first paragraph of section 3.2.

Good points made in respect of linking COS to SoF in Cochrane Reviews. Currently SoF are confined to a maximum of 7 outcomes. Some commentary on the implications of a COS for a particular condition/type of intervention having more than 7 domains is needed here.

> A benefit from considering a core outcome set in the design and registration of a review is that this might help reviewers to choose the outcomes to include in a Summary of Findings table. 75% of 300 published COS include 8 or fewer outcomes (manuscript under consideration). It is difficult to respond to this comment in general terms because the scope of a COS may be wider than the systematic review question.

Do the authors feel that Cochrane should insist on use of a COS if one exists, or at least justification of why it is not used?

> The use of COS by systematic reviewers is discussed in section 3.3.8.

In section 3.3.4, I feel that greater emphasis needs to be placed on there being an onus on developers to publish their COS (in much the same way as it is now expected that trial protocols be published) – as recognised above, dissemination is a necessary pre-requisite for implementation. Is there a case to be made for a specialist journal?

> The recommendation to make a COS study protocol publically available is made in section 2.4. Reporting guidance is described in section 2.14. COS need to be published in places accessible by COS users, which typically are likely to be in specialist journals for their discipline.

In section 3.3.6, once again a clearer distinction could be made between dissemination and implementation.

> The first paragraph of section 3.3.6 has been revised to reflect this point.

I felt that greater and earlier prominence needs to be given to the role of PPI in COS development and how this may be achieved. Funders increasingly require the selection of patient-centred outcomes, and a COS is surely more likely to be patient-centred if patients, carers are involved in its development.

> Section 2.6.1.2 addresses PPI in COS development.

It is not entirely clear to me what point is being made in the last paragraph on page 128 and throughout page 129. Is the argument about the need for periodic revision of COS or about the importance of PPI input to COS development.

> This text includes examples of planned COS reviews. A sentence has been added to explain this.

In section 3.5.2, consider suggesting that forms and checklists for peer reviewers explicitly remind them to consider whether the applicants have used a COS or justified why not.

> Recommendations for research, including methods to improve COS uptake, are given in Chapter 4.

Minor essential revisions

The first clause in the opening sentence seems odd – it is inconceivable that anyone would see the development of a COS as an end in itself, and indeed this is the conclusion drawn in section 3.6. Reworking of the opening sentence is required.

> The first sentence has been amended.

Funding bodies and journal editors need to be acknowledged as being amongst the actors at the foot of page 112.

> Text amended.

The sentence on page 128 “Given the low … gap” is not clear and needs reworking.

> Text amended.

Reviewer 3: John Norrie

Discretionary revisions

Chapter 1 - Introduction

Section 1.1 Line 1 – 'Human beings' – do the ideas of core outcome sets extend to pre-clinical studies involving animals? And to e.g. agricultural and industrial experiments?

> Yes, and there are examples of core outcome sets for the treatment of epilepsy in dogs for example. However COMET is focussed on clinical trials and we prefer to keep that focus from the start.

Section 1.1 bullet point 2. 'randomisation of persons to treatments' – is this general enough to include cluster designs?

> Text amended.

Section 1.1 Page 8 second para – I was a bit uncomfortable with the dogma about 'The primary outcome is the outcome considered to be of greatest importance to relevant stakeholders …' – for me, effectiveness studies struggle with this straight-jacket imposed by regulatory drug trials of a single clinical primary outcome. An effectiveness study has (at least) four dimensions – does it work, is it safe, is it acceptable, and at an affordable price. So the 'primary' outcome may well be different for different stakeholders, and it should be a considered assessment of all four that decides what treatments are carried forward.

Interestingly, you then seem to back away from the certainty of a single primary outcome by then saying (Page 9) that 'Harmful effects should always be viewed as important regardless of their primary or secondary outcome label' and then in the next paragraph acknowledge the importance of cost effectiveness '… for example the financial cost associated with that outcome' – and throughout Chapters 1 and 4 you emphasise the importance of patient involvement (hence the importance of acceptability of an outcome). So in an otherwise exemplary couple of chapters it struck a somewhat bum note that in an initiative designed to promote the best outcomes (to be harsh) you seemed to promote the uncritical adoption of a single primary outcome ('the one usually used in the sample size calculation')?

> Text amended.

Page 9. I wouldn't pick PSA as a good example of a surrogate end point! It has terrible properties, surely (at least as a screening measurement)?

> Text deleted.

1.2 Problems with outcomes. Foot of page 10 you finish describing patient-reported outcomes, top of page 11 immediately afterwards you describe line 3 'patient outcomes', which might be confusing. Suggest you just drop the 'patient' and say 'outcomes' in this latter sentence?

> Text amended.

Page 12 'meaning that on average a new instrument had been introduced for every fifth trial' – that looked a bit odd – it assumes that the introduction of new instruments is linear, which instinctively seems unlikely. I don't think you need this statistic, personally – point is well made.

> Text amended.

Page 13 Although I appreciate the 'reducing research waste' is an important initiative (e.g. Lancet series of publications) it is a bit negative in this context – the positive point would be that selecting the right outcomes would mean higher quality trials and getting reliable answers to improve patient health (either through primary research or meta-analyses) quicker? So it is more than 'reducing waste in research', surely?

> Text amended.

Page 18 Populating the COMET database – I didn't understand the purpose of the first paragraph 'Sinha and colleagues …' on paediatric trials? It seemed disconnected from the more general point made in the second paragraph about the systematic review of COS. Could you either integrate it / link it in or possible delete it?

> Text deleted.

Page 19 – 'correct as of Feb 2016' – that is over a year ago?

> This is due to the length of time from submission of the Handbook to review.

Page 20 – perhaps be more insightful about how COMET and PROMIS will endeavour to be compatible?

> PROMIS is concerned more with 'how' to measure patient-reported health status. Please see response to Reviewer 1 regarding the focus of this first version of the Handbook on the 'what' to measure.

Chapter 4 – Discussion

4.1. Recommendations for practice. Only COMET appears e.g. 'COS developers should register their project in the COMET database' without mentioning all the other initiatives – some of which seem relevant – discussed in Chapter 1. Should they be included or at least listed? The exception is COS-STAR but I wasn't sure what that was – was this introduced in Chapter 2 or 3?

> There is no other COS registry to our knowledge.

Page 135. I didn't understand the paragraph on 'If a Delphi study …' about feedback – it wasn't clear what the alternative was, for example. Suggest you rewrite this to make it clear what the point is?

> Text amended.

Page 136 1). I struggled a bit here on 'developers revealed that they would appreciated methodological guidance early on' and 'work is needed to compare existing methods in order to identify ways to minimise bias, maximise efficiency and increase uptake' – at first read it suggests in

an unquantified way that the currents sets of COS that have been developed may in some important respects be unfit for purpose? I don't think that this is what is intended?

> Chapter 2 describes the variability in methods used to develop COS. It is not yet known which methods may or may not be fit for purpose.

Page 138 This uncertainty is again underlined when the authors state 'To date, there has been no formal quality assessment of COS studies' – I think you need to either put in some quantified statement ameliorating this concern, or put it out front and say something like 'and of course such concerns on optimal methodology and quality of existing development work of COS may result in refinement or replacement of COS in future'.

> Any such comment would be too speculative at this point.

And interestingly on that point, I didn't see in an obvious way any discussion of how COS will be updated as e.g. measurement standards (e.g. the development of cheap and rapid imaging modalities in a clinical area) might influence the need to change a COS?

> This is covered in Chapter 3.

Also – and I might have missed it – is there any specific consideration of COS in screening and diagnostic studies, rather than clinical effectiveness studies – or is that out of remit?

> There are COS for screening trials in the COMET database.

Page 142 – I thought the material on Core Information Sets was fascinating but isn't this a massive issue in its own right? There is a huge amount of work going on around Informed Consent and Patient Information Leaflets just in the context of randomised trials – is it sensible to get into this here?

> COS for effectiveness trials are likely to overlap to some degree with core information sets and we felt this would be of interest to the reader.

Also, 'The importance of this issue has been highlighted in a recent landmark ruling by the Supreme Court' – I think you either have to give brief details (including which Supreme Court you are meaning) or perhaps just drop this?

> Text added.

Page 144 – it would have been useful to explain more fully (or exemplify) why a COS for routine care/quality measurement might differ from that for RCT. Naively, it is not obvious?

> This is the subject of current research and will feature in the next version of the Handbook.

Reviewer 4: Viktoria Eleftheriadou

Minor essential revisions

I have found a couple of typos in chapter 3. In particular:

Page 112: factors not actors

Page 116: 3.3 Implementation: Several factors (not actors)

Page 133: 3.6 Conclusions: Therefore, various factors (not actors)

> We do mean actors (not factors), i.e. participants in a process.

2nd round review

Reviewers 1-3: Accept - no further comments
